# *Blastocrithidia nonstop* mitochondrial genome and its expression are remarkably insulated from nuclear codon reassignment

**DOI:** 10.1093/nar/gkae168

**Published:** 2024-03-07

**Authors:** Dmitry A Afonin, Evgeny S Gerasimov, Ingrid Škodová-Sveráková, Kristína Záhonová, Ondřej Gahura, Amanda T S Albanaz, Eva Myšková, Anastassia Bykova, Zdeněk Paris, Julius Lukeš, Fred R Opperdoes, Anton Horváth, Sara L Zimmer, Vyacheslav Yurchenko

**Affiliations:** Faculty of Biology, Lomonosov Moscow State University, Moscow 119991, Russia; Faculty of Biology, Lomonosov Moscow State University, Moscow 119991, Russia; Institute for Information Transmission Problems, Russian Academy of Sciences, Moscow 127051, Russia; Life Science Research Centre, Faculty of Science, University of Ostrava, 710 00 Ostrava, Czechia; Department of Biochemistry, Faculty of Natural Sciences, Comenius University, 842 15 Bratislava, Slovakia; Institute of Parasitology, Biology Centre, Czech Academy of Sciences, 370 05 České Budějovice, Czechia; Life Science Research Centre, Faculty of Science, University of Ostrava, 710 00 Ostrava, Czechia; Institute of Parasitology, Biology Centre, Czech Academy of Sciences, 370 05 České Budějovice, Czechia; Department of Parasitology, Faculty of Science, Charles University, BIOCEV 252 50 Vestec, Czechia; Division of Infectious Diseases, Department of Medicine, University of Alberta, T6G 2R3 Edmonton, Alberta, Canada; Institute of Parasitology, Biology Centre, Czech Academy of Sciences, 370 05 České Budějovice, Czechia; Life Science Research Centre, Faculty of Science, University of Ostrava, 710 00 Ostrava, Czechia; Institute of Parasitology, Biology Centre, Czech Academy of Sciences, 370 05 České Budějovice, Czechia; Life Science Research Centre, Faculty of Science, University of Ostrava, 710 00 Ostrava, Czechia; Institute of Parasitology, Biology Centre, Czech Academy of Sciences, 370 05 České Budějovice, Czechia; Faculty of Science, University of South Bohemia, 370 05 České Budějovice, Czechia; Institute of Parasitology, Biology Centre, Czech Academy of Sciences, 370 05 České Budějovice, Czechia; Faculty of Science, University of South Bohemia, 370 05 České Budějovice, Czechia; De Duve Institute, Université Catholique de Louvain, 1200 Brussels, Belgium; Department of Biochemistry, Faculty of Natural Sciences, Comenius University, 842 15 Bratislava, Slovakia; University of Minnesota Medical School, Duluth Campus, Duluth, MN 55812, USA; Life Science Research Centre, Faculty of Science, University of Ostrava, 710 00 Ostrava, Czechia

## Abstract

The canonical stop codons of the nuclear genome of the trypanosomatid *Blastocrithidia nonstop* are recoded. Here, we investigated the effect of this recoding on the mitochondrial genome and gene expression. Trypanosomatids possess a single mitochondrion and protein-coding transcripts of this genome require RNA editing in order to generate open reading frames of many transcripts encoded as ‘cryptogenes’. Small RNAs that can number in the hundreds direct editing and produce a mitochondrial transcriptome of unusual complexity. We find *B. nonstop* to have a typical trypanosomatid mitochondrial genetic code, which presumably requires the mitochondrion to disable utilization of the two nucleus-encoded suppressor tRNAs, which appear to be imported into the organelle. Alterations of the protein factors responsible for mRNA editing were also documented, but they have likely originated from sources other than *B. nonstop* nuclear genome recoding. The population of guide RNAs directing editing is minimal, yet virtually all genes for the plethora of known editing factors are still present. Most intriguingly, despite lacking complex I cryptogene guide RNAs, these cryptogene transcripts are stochastically edited to high levels.

## Graphical Abstract

**Graphical Abstract:**
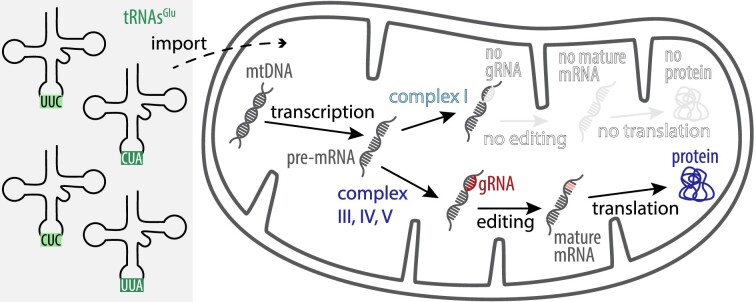

## Introduction

Protists of the class Kinetoplastea are famous for the ways in which their cellular and molecular processes differ from those of the ‘textbook eukaryote’ (1). These differences are shaped by often obligate parasitic lifestyles and a long independent evolutionary history (2–5). Notable deviations include massive *trans*-splicing as a part of RNA maturation (6), polycistronic transcription (7), and in one genus, extensive alteration of the canonical genetic code (8). While the latter is so far confined to the genus *Blastocrithidia*, the extent of its departure from the canonical code is extraordinary. All three stop codons of *Blastocrithidia* spp. have been recoded into sense codons with one of them, UAA, also used as the universal stop codon terminating translation. Moreover, the UGA codon is recognized by a highly unusual tRNA that allows a non-Watson-Crick base pairing with its anticodon (9). This departure from the near-universal genetic code and its implications for how tRNA molecules function in translation has placed a spotlight on *B. nonstop*, a model species of the genus *Blastocrithidia* (10,11). While the reasons for this wholesale recoding of the stop codons remain unclear, it may represent an efficient firewall shielding its bearer from viral infections and horizontal gene transfer (12). Fortunately, this protist is amenable to the study of variations, limitations, and genetic code re-writing as it: (i) is easily cultivable; (ii) has a streamlined, well-assembled and annotated genome and (iii) has a closely related sister genus *Obscuromonas* with a standard genetic code (9,13).

The mitochondrial genome of kinetoplastid flagellates, termed the kinetoplast DNA (kDNA), is composed of mutually catenated maxicircles and minicircles (14), and is highly divergent from organellar genomes of other eukaryotes. Specifically, on the maxicircle, the circular DNA molecule carrying protein-coding and ribosomal RNA loci, most genes are cryptically encoded (15). However, this is corrected on the RNA level *via* a byzantine uridine-insertion/deletion (U-indel) RNA editing pathway in which uridines (Us) are post-transcriptionally inserted and/or deleted (16–18). The overwhelming complexity of this process derives, in part, from the fact that hundreds of minicircle-encoded guide (g)RNAs are responsible for targeting hundreds to thousands of U insertions and dozens to hundreds of U deletions into specific sites to generate translatable open reading frames (ORFs) (19–21). Notably, kinetoplastid flagellates vary in the number and identity of their cryptogenes (loci producing transcripts that require editing). For example, mRNA encoding the NADH:ubiquinone oxidoreductase subunit 5 (*ND5*) of the electron transport chain (ETC) complex I requires editing in the free-living bodonid *Bodo saltans* (22), but not in trypanosomatids (23). Similarly, the ETC complex IV mRNA for cytochrome *c* oxidase subunit I (*COI*) is not edited in any species but *Trypanoplasma borreli* (24). Cryptogenes *ND8* and *COIII* encoding subunits of the ETC complexes I and IV, respectively, are often pan-edited, i.e. their transcripts are edited throughout except for the extreme termini. However, the genus *Wallacemonas* and the endosymbiont-containing subfamily Strigomonadinae represent exceptions in which the length of the edited domains is significantly reduced in the case of *ND8* or eliminated altogether in the case of *COIII* (25).

The complexity of RNA editing is also apparent in the 60+ proteins that either in complex or solo are part of the enzymatic, structural, and regulatory machinery necessary to execute these modifications (19,26). The RNA editing catalytic complex (RECC) and RNA editing substrate-binding complex (RESC) that comprise the bulk of the editing machinery are now much better understood than the genetic role of editing itself (17,27–29). RNA editing may also influence post-transcriptional regulation of gene expression in the absence of clearly identifiable gene-specific promoters on maxicircles and/or contribute to the variability and evolvability of kDNA (30,31). A final source of complexity is non-canonical editing events, i.e. the insertions or deletions of Us in positions and/or in multiples that are not consistent with an ORF. This contributes greatly to the challenge of organizing and describing U-indel edited transcriptomes. To address this, we developed a specialized toolkit called T-Aligner (20), which allows exhaustive mapping of edited reads (containing U-indels) to their cryptogenes of origin and efficient reconstructing of edited ORFs. Moreover, we have established a pipeline to map gRNA:mRNA interactions from minicircle sequences and RNA-seq reads, and to build complete gene editing maps (24,32).

Given the complexity inherent to mitochondrial U-indel RNA editing and other RNA processing steps in kinetoplastid flagellates, the *B. nonstop* mitochondrial genome and its expression appear vulnerable to perturbation (9). It is plausible to suggest that they are influenced by the extensive recoding of the nuclear genome in at least two ways. Firstly, nuclear genome codon reassignments may affect the structure and, thus, the function of the many mitochondrial proteins needed to process the mitochondrial RNAs or translate them. It has already been noted that *B. nonstop* nuclear-encoded mitochondrial ribosome proteins contain much higher number of in-frame stop codons than their cytosolic counterparts (9). Secondly, unique tRNAs that are present in the *B. nonstop* tRNA repertoire allow UAA and UAG to be read as glutamic acid, and the nuclear genome has accommodated this change. But, as a rule, kinetoplastids import all tRNAs necessary for mitochondrial mRNA translation from the cytosol (33–35). Thus, either the mitochondrial genome must undergo some sort of commensurate codon reassignment as the nuclear genome, or the mitochondrion of *B. nonstop* must specifically exclude tRNAs cognate to the stop codons either at the level of import or usage.

In this work, we characterize the mitochondrial genome and transcriptome of *B. nonstop* and analyze the suite of protein factors responsible for generating its mature coding transcriptome from precursor (and pre-edited) mRNAs. We specifically searched for evidence of accommodation of unique features of *B. nonstop*’s nuclear gene expression. In addition to demonstrating a clear delineation of codon usage between the nuclear and mitochondrial genomes and ruling out the most obvious mechanism to accommodate that change, we discovered additional unique features of the *B. nonstop* mitochondrial genome and its expression. We find the editing in this species to be simpler than in other, better-studied kinetoplastids, although virtually all the proteins known to play a role in the editing process remain conserved. We also document a tolerance for multiple short regions of amino acid (aa) insertions in mRNA processing proteins that may result from loss of components of a classical DNA repair pathway. Finally, we present evidence of high levels of apparently stochastic RNA editing of the complex I cryptogenes, despite the absence of any associated complex I-specific gRNAs.

## Materials and methods

### Species and cultivation

*Blastocrithidia nonstop* isolate p57 (9) was cultivated in Schneider's *Drosophila* medium (Thermo Fisher Scientific, Waltham, USA) supplemented with 2 μg/ml hemin (BioTech, Prague, Czechia), 25 mM HEPES pH 7.5, 100 units/ml of penicillin, 100 μg/ml of streptomycin (all from VWR, Radnor, USA), and 10% fetal bovine serum (BioTech) at 23°C with no shaking in vertical flasks. For nucleic acid purification and enzymatic assays, *B. nonstop* was harvested at mid-log phase of growth, a density of 2 × 10^7^ cells/ml. *Phytomonas serpens* isolate 9T (36) was cultivated at 23°C in Brain Heart Infusion medium (BD, Franklin Lakes, USA) supplemented with 10μg/ml hemin (PanReac AppliChem, Darmstadt, Germany). Species identity was confirmed as previously (37,38).

### Nucleic acid purification and sequencing

Illumina sequenced reads from a previously published total DNA sequencing library (library 1) (9), one new kDNA library (library 2), and four RNA libraries were used in this study (described in Supplementary Table S1 along with library preparation information and sequencing protocols). Two of the RNA libraries were biological replicates of poly(A)-enriched total RNA libraries (libraries 3 and 4). One RNA library was a kinetoplast RNA (kRNA)-enriched RNA library (library 5), and the final library was a small RNA library with kRNA-enriched material used as input (library 6). Neither of the two latter libraries was poly(A)-enriched. For total RNA libraries, RNA was purified from 5 × 10^7^ cells using TRIzol reagent (MRC, Cincinnati, USA) and was subsequently treated with DNase I (New England Biolabs, Ipswich, USA) according to the manufacturer's protocol. The RNA was further purified with the Direct-zol RNA miniprep Plus kit (Zymo Research, Irvine, USA). The kRNA was obtained from mitochondrial vesicles isolated as described previously (20), except that 5 × 10^10^ cells were used as starting material. RNA was isolated and processed as above. RT-qPCR quantification of the *ND1* transcript (20) was used to verify that the sample was enriched for kRNA. For the small RNA library, mitochondrial vesicles were purified from 1.7 × 10^10^ cells and small RNAs were processed as previously (32). Library preparation and sequencing were done at Macrogen Europe (Amsterdam, the Netherlands) for kDNA and total RNA libraries, and the Institute of Applied Biotechnologies (Olomouc, Czechia) for kRNA and small RNA libraries. Sequencing data are deposited at NCBI under the SRA accession numbers specified in Supplementary Table S1. No libraries were discarded and all raw reads were utilized for downstream analysis (reads deriving from the nuclear genome were not removed).

### Assembly and analysis of mitochondrial genome components

The full sequence of the *B. nonstop* maxicircle was extracted from the genome assembly reported earlier (9) and polished with new kDNA-derived Illumina reads (library 2, Supplementary Table S1) using Pilon v. 1.2.4 (39). Maxicircle genes were manually annotated using maxicircle-encoded proteins of the trypanosomatids *T. cruzi* and *L. pyrrhocoris* (32,40) as references with NCBI Translation Code 4 (mold, protozoan and coelenterate mitochondrial). Approximate annotations of cryptogene boundaries were later refined for *A6* subunit of ATP synthase, *COII*, *COIII*, apocytochrome *b* (*CYb*) and ribosomal small subunit protein 12 (*RPS12*) using the ORF ends determined with the T-Aligner ORF prediction module (20) as they became available (see below).

Minicircles were assembled from kDNA-derived paired-end Illumina reads obtained specifically for this study (library 2, Supplementary Table S1) using isolate and metagenome modes of SPAdes v. 3.15.4 (41,42). Initially, minicircle-like contigs were extracted by searching for the typical Conserved Sequence Block (CSB) 3 pattern, ggggttggtgtg (43,44). Obtained contigs with terminal overlapping sequences were circularized. Motif finding in the minicircle sequences was done with MEME suite v. 5.5.1 (45). Several minicircle contig candidates were additionally extracted from the SPAdes output using the CSB1 palindromic hairpin (yyryryrrrryyyyryryrr) of *B. nonstop* identified by MEME suite. All 41 minicircles were detected in total DNA sequencing data (library 1, Supplementary Table S1) by read mapping and minicircle contig coverage assessment. *De novo* assembly using the same total DNA library yielded no new minicircle sequence classes. Consequently, the kinetoplast genomes of *B. nonstop* in the previous and current study did not differ in the repertoire of minicircle classes. Minicircle inverted repeats were detected by EMBOSS einverted v. 6.6.0 (46) with the following options: ‘-gap 14 -match 4 -threshold 40′. The assembled mini- and maxicircle sequences were submitted to GenBank under accession numbers OQ909994–OQ910035.

### Assembly and analysis of mitochondrial transcriptome

Both poly(A)-enriched total RNA libraries (libraries 3 and 4, Supplementary Table S1) and the kRNA-enriched library (library 5, Supplementary Table S1) were independently mapped to the maxicircle using Burrows-Wheeler Aligner (BWA) v. 0.7.17 (47) and alignments were processed with SAMTools v. 1.17 (48) and BEDTools v. 2.30.0 (49) to produce initial RNA coverage profiles using unedited reads only. T-Aligner v. 4.0.5f (24) was used to reconstruct cryptogene ORFs and capture levels of both edited and unedited mapped reads across the maxicircle coding region as reported previously (40). To assemble the cryptogene *A6* specifically, reads of the small RNA library (library 6, Supplementary Table S1) were added to the other RNA libraries and the search depth was increased to its maximum level. The mRNA sequences were submitted to GenBank under following accession numbers: OQ911728 (*COIII*), OQ911729 (*COII*), OQ911730 (*CYb*), OQ911731 (*A6*) and OQ911732 (*RPS12*).

Minicircle- and maxicircle-encoded gRNAs were initially annotated using ‘findgrna’ tool from T-Aligner that predicts gRNA coding genes with a minicircle:mRNA alignment procedure used previously (24,40).

To identify gRNA reads among those of the small RNA sequencing library, the reads were first trimmed with Trimmomatic v. 0.39 (50) with ILLUMINACLIP and MINLEN:15 options to remove adapter sequences and very short reads. Completely overlapping read pairs from trimmed reads (representing captured gRNA molecules that are shorter than the read lengths) were merged with BBMerge v. 39.0 (51). They were mapped onto the mini- and maxicircle using BWA. Sorted BAM files were generated with SAMTools. Mini- and maxicircle-aligned merged reads deemed likely to be derived from the gRNA population were subsequently utilized to determine each gRNA’s precise termini based on the consensus of multiple reads and after removal of post-transcriptionally added 3′ nucleotide extensions. This was executed by extracting them separately, removing poly(T) and poly(A) post-translationally added nucleotide tails with custom bash scripts (available at the T-Aligner GitHub page), and clustering them by CD-HIT v. 4.8.1 (52) with an identity threshold set at 97%. The consensus sequences from obtained clusters were considered possible gRNAs and validated as such if they mapped back to the minicircles or maxicircle. Boundaries of these ‘functional’ gRNA genes were marked on the minicircles. The final minicircle sequences were re-orientated to start with the CSB block closest to the functional gRNA gene.

For identification of putative tRNAs in the mitochondrial genome, tRNAscan-SE v. 2.0.11 (53) in ‘organellar’ mode and ARAGORN v. 1.2.38 (54) in ‘standard’ and ‘metazoan mitochondrial’ modes were used. The ARAGORN-predicted tRNA-like structures were further analyzed by tRNAscan-SE On-line tool (55).

### Editing cascade reconstruction for canonical ORFs

RNA editing cascade maps of successive gRNA usage were generated using gRNA:mRNA alignments. Previously reconstructed canonically edited mRNAs for cryptogenes *RPS12*, *COIII* and *A6* and the set of 41 minicircle sequences were used as input for the ‘findgrna’ tool from T-Aligner suite. Obtained alignments were filtered using the following criteria: (i) alignments that utilized minicircle loci outside of the refined gRNA gene boundaries were discarded, (ii) alignments with three or more adjacent mismatches were discarded, (iii) alignments with mismatch/length ratio >1/8 were discarded. Minicircles were subsequently sorted according to the order in which their gRNAs edit the main pathways of the *RPS12*, *COIII* and *A6* cascades.

### Analysis of potential guiding of all editing events observed in read libraries

Raw sequencing reads from the two poly(A)-enriched total RNA libraries were mapped on all putative maxicircle cryptogenes with T-Aligner's ‘alignlib’ tool; output TAF files were processed by custom python script (available at the T-Aligner GitHub page) and reads with ten or more U insertions and deletions total were extracted for further analysis. The set of 41 discovered gRNAs was aligned to the extracted reads with the ‘findgrna’ tool from the T-Aligner suite with a relaxed search setting: ‘–seed_score 20 –seed_length 16 –length 21 –score 25 –gu 17 –mm 4 –anchor 2′. Read:gRNA alignments were grouped by cryptogenes. Distributions of G:U pairs, mismatches and alignment length were determined in groups of alignments for gRNAs involved in editing of *A6, RPS12* and *COIII* and editing reads mapped on *ND3*, *ND8*, *ND9*, *G3* and *G4*.

### Organellar fractionation and tRNA localization using northern blotting analysis

A total of 2 × 10^8^, 3 × 10^8^ and 4 × 10^8^ cells were harvested by centrifugation, washed in PBS, resuspended in 500 μl SoTE (600 mM Sorbitol, 2 mM EDTA, 20 mM Tris–HCl pH 7.5) and lysed with 500 μl SoTE containing 0.1% digitonin for 5 min on ice before centrifugation (4°C, 8,000 × g, 5 min). The supernatant was treated as the cytosolic (C) fraction. The pellet was resuspended in 500 μl SoTE supplemented with 2 μg/ml RNaseA, 2 units DNase I (New England Biolabs), 3 mM MgCl_2_ and incubated on ice for 15 min. The resultant pellet after centrifugation (as above), was treated as the mitochondrial (M) fraction.

RNA was isolated using the guanidinium thiocyanate/phenol/chloroform extraction method (56). RNA samples (2 μg each) were separated on denaturing 8% polyacrylamide gel with 8 M urea, transferred onto Zeta-probe membranes (Bio-Rad Laboratories, Hercules, USA), and probed with ^32^P-labeled oligonucleotides specific for each RNA as follows: tRNA^Glu^UUA: 5′-gtcgcctgggttaaagccaga-3′; tRNA^Glu^CUA: 5′-gaatggcgggttagagccgcg-3′; tRNA^Glu^UUC: 5′-atttcctgggtgaaagccagg-3′; tRNA^Glu^CUC: 5′-ttccggtgccggggatcgaac-3′; 5.8S rRNA: 5′-attgggcaatgaaatgattctg-3′; 9S rRNA: 5′-acggctggcatccttttc-3′. Images were captured using a Storm PhosphorImager 860 (Molecular Dynamics/ GE HealthCare, Chicago, USA). The mitochondrial signal (M) abundance relative to the total signal (C + M) was calculated as a percentage.

### In-gel staining of complex I

Trypanosomatid mitochondria were isolated as described previously (57). Mitochondrial protein lysates were obtained from pellets by incubating them in 0.5 M aminocaproic acid and 2% (w/v) dodecyl maltoside (both PanReac AppliChem) for 30 min on ice; the supernatant was further centrifuged at 20,000 × g at 4°C for 30 min. For each species, 100 μg of mitochondrial lysates were separated by high resolution 2–10% (w/v) clear native polyacrylamide gel electrophoresis (58). The gel was incubated in 100 mM Tris–HCl pH 7.4, 140 μM NADH (Merck, Darmstadt, Germany), 1 mg/ml nitrotetrazolium blue (PanReac AppliChem) to detect NADH dehydrogenase activity of complex I. Total protein content in gels was determined by staining with Coomassie Brilliant Blue (PanReac AppliChem).

### Identification of nucleus-encoded subunits of ETC and mitochondrial RNA-processing enzymes

Seventy-five mitochondrial RNA processing enzymes identified previously in *Trypanosoma brucei* TREU927 (19), or their orthologues in *Leishmania major* Friedlin (obtained from the TriTrypDB, release 66 (59)) and 30 nucleus-encoded components of complexes I and V (60,61), were used as queries for BLASTp and tBLASTn v. 2.13.0 searches in the genome-derived proteome and genome of *B. nonstop* (9), respectively (Supplementary Table S2 and S3). The validity of forward hits was confirmed by reciprocal BLAST searches against the *T. brucei* proteome.

Maximum likelihood phylogenetic analyses were conducted to distinguish the *B. nonstop* KREPB6 and KREPB7, and to verify that duplication of RESC11 and RESC12 was restricted to *T. brucei* and close relatives. Protein orthologues were retrieved from the TriTrypDB. Sequences were aligned by MAFFT v. 7.508 (62) with L-INS-I algorithm and trimmed by trimAl v. 1.4 (63). The phylogenetic trees were inferred using IQ-TREE v. 2.2.0 (64) under the JTT + F + G4 (RESC11/12) and JTT + I + G4 (KREPB6 and 7) models, which were determined as best-fitting according to Bayesian information criterion. Branch supports were estimated with 1,000 ultrafast bootstrap replicates.

Putative cytidine deaminases and glutamyl-tRNA synthetase were analyzed previously (65,66) and identified in the *B. nonstop* data as above. Possible mitochondrial targeting signals were identified by TargetP v. 2.0 (67), MultiLoc2 in animal and fungal settings (68), Predotar (69), TPpred3 (70) or by an in-house search of the following pattern: ^M-[RHKFL]-x{0,1}-[RKHST]-x{1,10}-[STRK] as described previously (71).

The usage of standard and non-standard glutamate and tryptophan codons was analyzed as described previously (8).

### Protein modeling

Swiss-Model (72) was utilized to model complete *B. nonstop* RESC-A and RESC-B complexes and their individual subunits using *T. brucei* cryo-electron microscopy (cryo-EM) structures (26) as templates. To ensure that homology models were not biased towards the templates in localized regions, RESC-A complex and its subunits were also modelled with AlphaFold v. 2.3.0 (73) using a sequence database customized for trypanosomatids (74). Swiss-Model was also used for homology modelling of COI, ND1, ND2, ND4 and ND5 from both *B. nonstop* and *T. brucei*.

## Results

### *Blastocrithidia nonstop* maxicircle genes reveal coding sequence erosion

**Figure 1.**
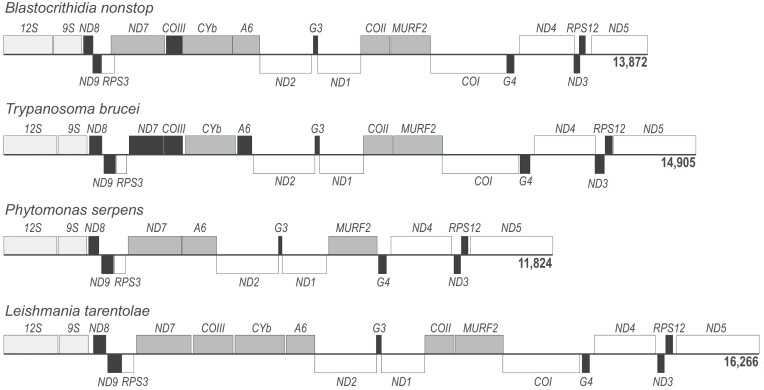
Maxicircle coding regions for four evolutionarily distant trypanosomatid species: *Blastocrithidia nonstop*, *Trypanosoma brucei*, *Phytomonas serpens* and *Leishmania tarentolae*. The top and bottom boxes indicate genes encoded on different strands. Ribosomal RNAs – patterned, unedited genes – white, cryptogenes edited at only a short domain (minimally-edited) – grey, pan-edited cryptogenes – black. For *B. nonstop*, a locus was described as a cryptogene if the sequence would require editing to generate an open reading frame, independent of the presence or absence of edited reads in sequencing libraries. Several loci are alternatively named in maxicircles of different species: *RPS3* (*MURF5*, *uS3m*), *RPS12* (*uS12m*), *ND2* (*MURF1*), *G3* (*ND4L*, *CR3*), *G4* (*ND6*, *CR4*), *ND3* (*CR5*) (85,102,103).

We searched for maxicircle encoded genes with the standard protistan mitochondrial genetic code (in which UGA encodes tryptophan (75)) and identified *COI*, *ND1*, *ND2*, *ND4*, *ND5* and *RPS3*. *Blastocrithidia nonstop* maxicircle genes are syntenic with those of other investigated trypanosomatids, and its coding region (CR) length of 13,872 bp is within the range of other species (76–79) (Figure 1). The CR boundaries and termini of some genes and cryptogenes do vary somewhat from those of other sequenced maxicircles, as illustrated by genes that do not require editing. The translated product of *ND2* has an N-terminal truncation and short gaps of 6–21 aa compared to other aligned kinetoplastids, and a similar erosion of ND4 and ND5 is also apparent (Supplementary Figure S1). The aa sequences of *in silico* translated mitochondrial transcripts share less identity with homologues of related species than expected. For example, *ND1* and *COI* genes encoding subunits of complexes I and IV, respectively, have lengths similar to their homologues of other trypanosomatids, but their protein products display a marked loss of aa identities (Supplementary Figure S1). *Blastocrithidia nonstop*’s maxicircle gene products are even more divergent in aa identity than those of *P. serpens*, whose maxicircle possesses extensive CR alterations (Figure 1 and Supplementary Figure S1) (77). Regardless of their sequence-level divergence, homology modelling of *B. nonstop* COI, ND1, ND4 and ND5 returned structures fully compatible with their expected canonical folds. While utilization of the putative *B. nonstop* ND2 as a Swiss-Model query did not return any of the many available ND2 structures as a template, neither did *T. brucei* ND2. Overall, despite apparent coding sequence erosion, the proteins encoded by the *B. nonstop* maxicircle appear compatible with their conventional function.

### *Blastocrithidia nonstop* maxicircles do not encode tRNAs

The first step in evaluating mitochondrial codon usage was to assemble the *B. nonstop* maxicircle utilizing previously published data (9) and polishing the assembly with newly-generated kDNA-enriched reads (library 2, Supplementary Table S1). In doing so, we also verified that, as with other kinetoplastids, nuclear tRNAs are the only source of tRNAs for the organellar translation in *B. nonstop*. Two algorithms were used to scan the mitochondrial genome for tRNAs. While the ARAGORN predicted tRNA-like conformations in the maxicircle, all of them exhibited substantial deviations in critical tRNA components and their identity was not confirmed by the tRNAscan-SE online tool. Thus, as expected, translation of the *B. nonstop* mitochondrial mRNAs is entirely dependent on nucleus-encoded tRNAs.

### *Blastocrithidia nonstop* utilizes a standard protistan mitochondrial genetic code

With the maxicircle CR of *B. nonstop* characterized, we next determined the products of *B. nonstop* U-indel RNA editing by reconstructing translatable, edited mRNAs for each expected maxicircle cryptogene locus using merged RNA libraries and T-Aligner (Figure 1, all loci shaded black or gray). Edited ORFs of expected sizes were successfully reconstructed only for the lone maxicircle-encoded complex III subunit *CYb*, the two complex IV subunits that require editing (*COII* and *COIII*), the mitoribosomal protein *RPS12* and the *A6* subunit of ATP synthase. All these transcripts are also edited in *Trypanosoma* spp. (40,80,81). We then verified that both standard protistan mitochondrial stop codons, UAA and UAG are used in *B. nonstop*. Of its six correctly encoded mRNAs *(ND1*, *ND2*, *ND4*, *ND5*, *COI* and *RPS3*) and five edited mRNAs (*A6*, *COII*, *COIII*, *CYb* and *RPS12*), eight and three use the UAA and UAG termination codons, respectively. Thus, there appears to be no major recoding of the *B. nonstop* mitochondrial genome.

### *Blastocrithidia nonstop* does not appear to exclude unique read-through tRNAs from the mitochondrion

**Figure 2.**
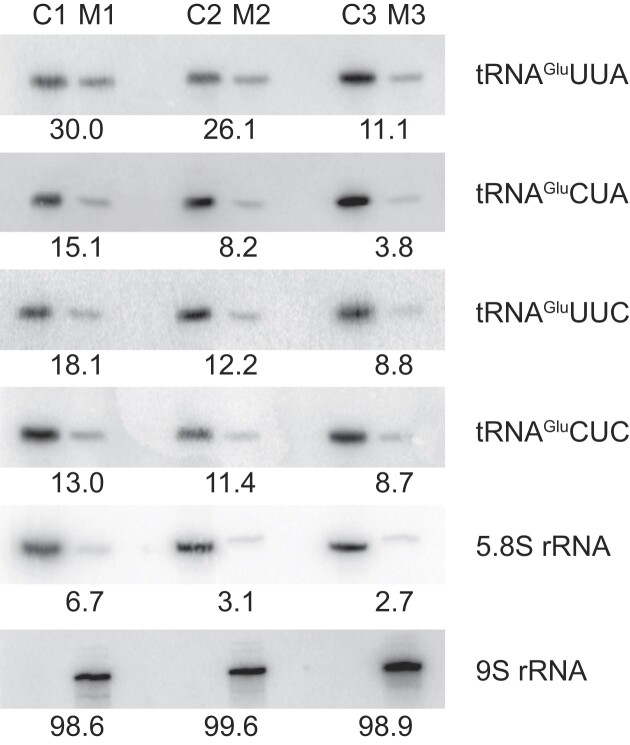
tRNAs^Glu^ import into the *B. nonstop* mitochondrion. Cytosolic (C1, C2 and C3) and/or mitochondrial (M1, M2 and M3) RNA was isolated from 2 × 10^8^, 3 × 10^8^ and 4 × 10^8^ cells, respectively, and probed with radiolabeled oligonucleotides specific for a given RNA. The 5.8S and 9S rRNAs were used as quality controls to assess purity of the cytosolic and mitochondrial fractions, respectively. The number under each blot indicates the percentage of mitochondrial (M) relative to the total (C + M) signal.

Some apparatus must exist to prevent erroneous read-through of UAA and UAG stop codons of mitochondrial mRNAs. A potential mechanism to avert this is to exclude the two read-through tRNA^Glu^ from the mitochondrion entirely, similarly to tRNA^Sec^ and tRNA^Met-I^ in other trypanosomatids (35,82,83). This would be inherently efficient as these tRNAs would not serve any apparent purpose in the mitochondrion. We tested this by northern blotting analysis after purifying mitochondria from *B. nonstop* and comparing the abundance of canonical (UUC and CUC) and suppressor (UUA and CUA) tRNAs^Glu^ in the mitochondrial extract (labeled M in Figure 2) to that of the total cellular material (quantification in Figure 2). While it is nearly impossible to completely fractionate mitochondria from other cellular components, we at least expected that the fraction of the suppressor tRNAs^Glu^ in mitochondrial extract would be less than that of the needed canonical tRNAs^Glu^ and approximately equal to the signal from the 5.8S rRNA used to track cytosolic contamination in the mitochondrial fraction. This would indicate an active import of the canonical tRNAs^Glu^ into the mitochondrion and a retention of the suppressor tRNAs^Glu^ in the cytosol. Instead, we found that mitochondrial fraction signal of the read-through tRNAs^Glu^ was always stronger than that of the 5.8S rRNA. In fact, mitochondrial fraction signals for the suppressor tRNAs^Glu^ were typically stronger than those of the canonical tRNAs^Glu^ suggesting that rather than being excluded, the read-through tRNAs are entering the mitochondrion at equivalent or greater rates than the canonical tRNAs. Relative to what is observed in *T. brucei*, the *B. nonstop* tRNA import machinery appears to be less selective.

**Figure 3.**
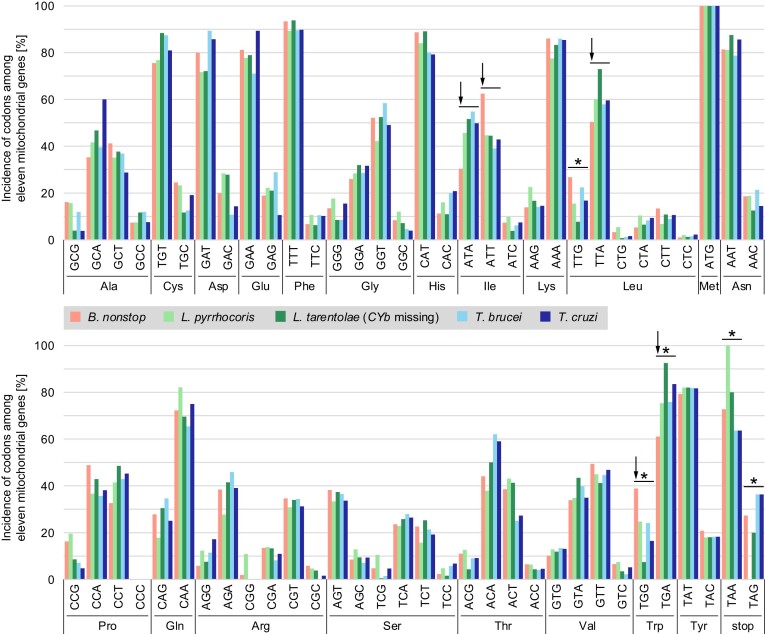
Analysis of codon usage in *B. nonstop* maxicircle-encoded mRNAs. Incidence of codon usage among eleven mitochondrial mRNAs. Arrows indicate codons for which incidence in *B. nonstop* appears to differ from that of other examined species. Asterisks indicate codons for which incidence in some other species appears to specifically differ from that of other species.

A relatively nonselective *B. nonstop* tRNA import mechanism compared to other kinetoplastids could possibly result in mitochondrial codon usage that differs from that of other species. To analyze that, we compared the overall codon usage of its eleven translatable mRNAs to that of the same mRNAs of other trypanosomatids (Figure 3). While some variability is apparent, we documented multiple instances of specific codon over- and under-utilization in *B. nonstop* relative to all other species examined (Figure 3, arrows). In one instance, lesser utilization of one codon corresponded to greater utilization of another codon of the same amino acid (tRNA^Ile^, compare ATA to ATT). However, there were occasional aberrancies in specific codon incidence in other species as well, specifically two instances of underutilization in *L. tarentolae* and one instance of overutilization in *L. pyrrhocoris*. (Figure 3, asterisks). Therefore, our results suggest that tRNA import strategies may not be consistent across kinetoplastids. Of course, the question remains as to how the mitochondrial genome remains apparently unaffected by codon reassignment of the nuclear genome given these findings.

### Cryptogenes of the complex I are transcribed but not productively edited in *B. nonstop*

T-Aligner failed to reconstruct edited ORFs for all identified complex I subunit cryptogenes (*ND3*, *ND7*, *ND8*, *ND9*, *MURF2*, and the putative *ND4L* and *ND6* homologues *G3* and *G4*, respectively (84,85)). To the best of our knowledge, their transcripts are edited in all investigated trypanosomatids, although corresponding genes are absent in the distantly related kinetoplastid *Trypanoplasma borreli* (24).

**Figure 4.**
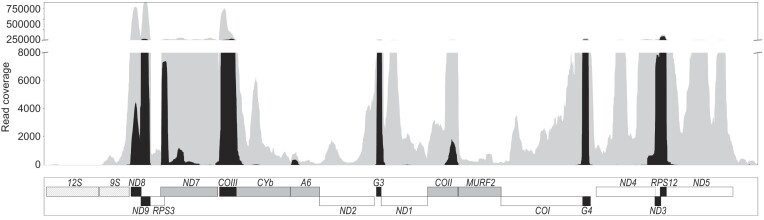
Transcription of *B. nonstop* maxicircle genes. The number of detected reads is shown on the Y axis in grey. The number of edited reads (reads ≥5 edited sites) is shown in black. The schematic organization of the maxicircle CR (the greyscale scheme corresponds to that in Figure 1) is shown below.

To determine if complex I genes and cryptogenes were even expressed, we determined the maxicircle expression profile. All maxicircle genes and putative cryptogenes appear to be effectively transcribed, including those of complex I (Figure 4). The maxicircle expression profile was similar regardless of the library used for its generation, with the number of edited reads substantially lower in the kRNA-libraries generated without poly(A) selection (Supplementary Figure S2). The major transcription peaks were of genes *ND8*, *ND9* and *COIII*, similar to the pattern observed in several *Trypanosoma cruzi* strains (40). As expected, pan-edited *RPS12* and *COIII* were well-covered with both edited and pre-edited reads. Surprisingly however, ample reads possessing U insertions and deletions relative to the maxicircle sequence also mapped to all the edited complex I transcripts but *MURF2* (Figure 4) despite the absence of discernable patterns of editing. This paradox will be parsed by analysis of the gRNA population presented below.

### Experimentally confirmed loss of complex I activity in *B. nonstop*

**Figure 5.**
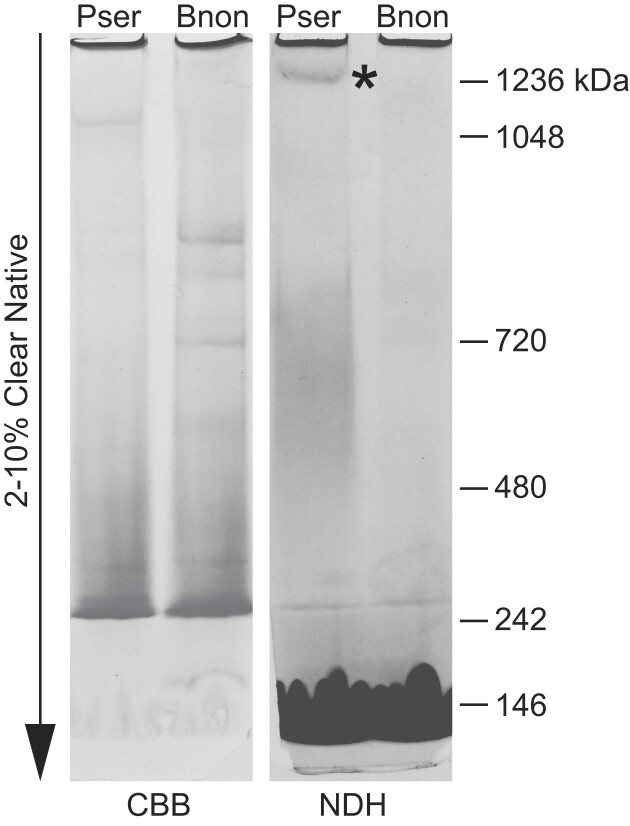
Electrophoretic analysis of the activity of complex I in mitochondrial lysates of *P. serpens* and *B. nonstop*. Mitochondrial proteins were stained by Coomassie Brilliant Blue in 2–10% Native gel (CBB). Complex I was visualized by the in-gel activity assay of NADH dehydrogenase (NDH). The asterisk indicates a position of the missing activity band of complex I in *B. nonstop*. Sizes of molecular weight marker bands (in kDa) are indicated on the right.

We wished to verify the loss of complex I, the largest respiratory chain enzymatic complex, at the protein level. Despite the fact that genes for its subunits are present in all the trypanosomatid species studied thus far, this complex is fully functional in only three of the eleven Trypanosomatidae spp. investigated in detail (57,60,86). Although we identified nucleus-encoded subunits of complex I in the *B. nonstop* genome (Supplementary Table S3), we could not deduce their functionality because of the presence of in-frame stop codons. In line with this, only two nucleus-encoded complex I subunits were identified in the proteomic data of *B. nonstop* (9). These proteins, NDUFS5 and NDUFAB1 also act in other pathways unrelated to complex I (60,85), and the gene encoding NDUFAB1 is also present in the *Trypanoplasma borreli* genome that lacks mitochondrial complex I genes entirely (24). In contrast, eight of nine complex V subunits encoded in the nucleus were identified in the same proteomic dataset (Supplementary Table S3). Finally, we analyzed whether the activity of an intact complex I could be detected by native PAGE in-gel activity assays. In contrast to *P. serpens* that was used as a positive control, in the mitochondrial lysate of *B. nonstop* there was no NADH dehydrogenase activity at the high molecular weight region where the intact complex I typically migrates (Figure 5). While the missing activity should not be overinterpreted (complex I may be difficult to detect (87)), its apparent absence strengthens our conclusion that complex I is absent in *B. nonstop*.

### The complete repertoire of *B. nonstop* minicircle classes is limited

**Figure 6.**
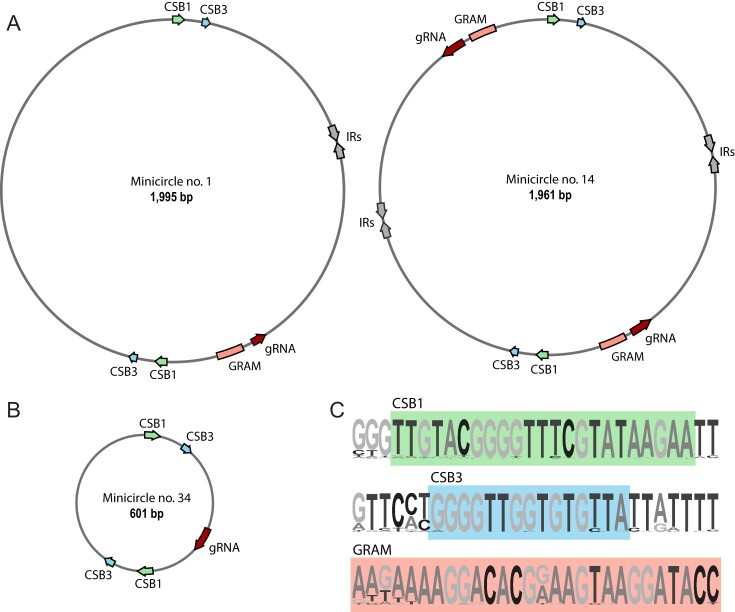
*Blastocrithidia nonstop* minicircles. (**A**) Minicircles of classes 1 and 14 that serve as representative examples of the main size group. All molecules have a gRNA gene and a gRNA-associated motif (GRAM) on the opposite strand of that of the conserved sequence blocks (CSBs). IR, inverted repeat. (**B**) Minicircle of class 34, which substantially differs in size from all other minicircle classes and encodes a single gRNA. (**C**) Block sequence logos for CSB1, CSB3 and GRAM. Conserved motifs are boxed.

Next, we focused on discovery of a mechanism to explain the lack of translatable mRNA products of any complex I subunit cryptogenes. The majority of kDNA consists of minicircles that encode gRNAs responsible for directing U-indel editing. It is possible that the observed phenomenon was caused by the absence of gRNAs needed to direct editing of complex I subunit cryptogene transcripts. To determine whether the *B. nonstop* minicircles encode such gRNAs, we first assembled its minicircle repertoire *de novo* and verified the presence of 41 identified minicircle classes (referred to hereafter simply as ‘minicircles’) in previously sequenced (9) and current datasets (Supplementary Table S1). The sizes of 40 of the 41 minicircles range between 1920 and 2019 bp (represented by classes 1 and 14 in Figure 6A), while class 34 minicircles (Figure 6B) are only 601 bp long. All *B. nonstop* minicircles (including class 34) resemble the ‘dimeric’ *L. pyrrhocoris* minicircles (32) with two regions of high conservation that are oriented head-to-tail and positioned at opposite poles. Conserved regions include CSBs 1 and 3 (44) (Figure 6C). We identified inverted repeats in each minicircle except for those belonging to the class 34. As in the case of the maxicircle, the same tRNA search algorithms run on this minicircle population uncovered only a few degenerate tRNA-like structures.

Strikingly, 41 minicircle classes is the lowest number documented in any trypanosomatid species investigated thus far. For example, the minicircle repertoires of *Trypanosoma lewisi*, *L. pyrrhocoris* and *T. brucei* include 58, 67 and 391 classes, respectively (21,32,81). Unlike some other strains with low minicircle diversity, the analyzed isolate of *B. nonstop* has existed in continuous cultivation for a relatively short period of time, reducing the possibility of extensive minicircle loss due to prolonged cultivation (88,89). Regardless of how it came about, *B. nonstop* low minicircle diversity suggests that its ability to edit the maxicircle transcriptome is limited.

### Loss of editing for complex I subunit transcripts in *B. nonstop* is traced to loss of gRNAs

To determine whether identified minicircles are insufficient to encode the necessary gRNAs for complex I subunit mRNA editing, we first identified gRNAs capable of directed editing of portion(s) of edited mRNA in T-Aligner-identified *CYb*, *COII*, *COIII*, *A6* and *RPS12* on either the *B. nonstop* minicircles or the maxicircle. We found three gRNA loci in the maxicircle, easily detectable because they mirror the identity and position of those located in the *L. pyrrhocoris* maxicircle (32). These gRNAs completely satisfy the guiding requirements for the limited edited domains of *COII* and *CYb* cryptogenes (Supplementary Figure S3). To find gRNAs for *COIII*, *A6* and *RPS12*, we aligned edited maxicircle genes to minicircles using an established pipeline (24,32). Unlike the *L. pyrrhocoris* minicircles which are of about the same size and usually contain two gRNAs *per* molecule or the *Trypanosoma* spp. minicircles that encode three or four gRNAs (81), we detected only one putative gRNA on each of the 40 minicircle classes and two gRNAs on a minicircle of the class 14 (Figure 6). Once these gRNA loci were mapped (Supplementary Table S4, tab Minicircles), a conserved ‘gRNA-associated motif’ (labeled GRAM, Figure 6) was identified near gRNA-encoding genes in all minicircles except for the unusually small class 34 minicircle. Using small RNA sequence libraries, we confirmed that all edited ORF alignment-identified putative gRNAs are expressed. The minicircles of the classes 40 and 41 lacked regions of alignment with the T-Aligner reconstructed edited ORFs. However, a single gRNA locus on each of them was identifiable through alignment with small RNA sequence library reads (Supplementary Table S4).

**Figure 7.**
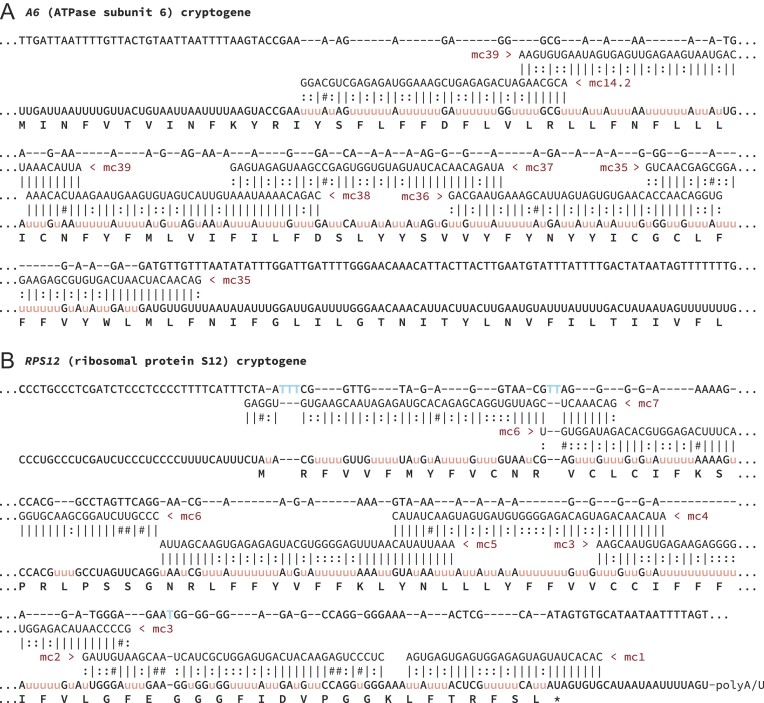
Reconstruction of the cascade of gRNAs required to direct editing of the pan-edited *A6* and *RPS12* cryptogenes. The top line in each panel represents the cryptogene DNA sequence. Under the DNA line, the gRNA sequences (in 3′-5′ direction) are displayed, aligned to the corresponding positions of the mature mRNA. Inserted and deleted nucleotides are shown in light-red lowercase (u) and blue (T), respectively. Vertical lines ‘|’, colons ‘:’ and hashes ‘#’ in alignments represent Watson–Crick pairs, G:U pairs and other mRNA:gRNA interactions, respectively. The label ‘mcN’ refers to an aligned gRNA, where *N* is the number of minicircle encoding a particular gRNA (Supplementary Table S4). The encoded amino acid sequence is presented under the row of mature mRNA (at the bottom of each panel).

To determine whether the identified gRNA repertoire could account for all the editing needed to generate translatable ORFs on the three identified pan-edited mRNAs, we reconstructed their predicted gRNA usage. All 40 relevant gRNAs mapped with sequential coverage across the pan-edited regions. Apart from two positions on *COIII* with gRNAs that were adjacent but not overlapping, gRNAs largely appear sufficient to direct editing of the pan-edited transcripts. However, there was virtually no redundancy of coverage when considering the overlap thought to be essential for gRNA ‘anchor region’ binding (Figure 7 and Supplementary Figure S4). A lack of redundancy is atypical of what is usually observed. Notably, as all identified gRNAs must be utilized to generate the translatable *COIII*, *A6* and *RPS12* transcripts, there are only two unaccounted *B. nonstop* gRNAs.

Not only does T-Aligner fail to reconstruct edited ORFs for *G3*, *G4*, *ND3*, *ND8* and *ND9*, two unassigned gRNAs would be wholly insufficient to guide their productive editing. Nevertheless, many reads mapping to these cryptogenes were edited. Thus, the emerging question was: what is the source of their apparently nonproductive editing? Potentially, the binding of anchor regions of cognate gRNAs for *RPS12*, *COIII*, *A6*, *CYb* or *COII* to the complex I subunit transcripts may result in editing to a specific but nonproductive pattern on the complex I subunit transcripts. To explore this possibility, we aligned all identified gRNAs to reads mapping to complex I cryptogenes that contained ≥10 editing sites. This default value is consistent with editing typically guided by more than one gRNA. The gRNA:read alignments demonstrated that non-cognate binding and subsequent guiding by the known gRNAs could result in most of the observed editing events (79–86% depending on the cryptogene, which is close to the values observed for *L. pyrrhocoris* (32)). As these putative guiding events do not result in patterns contributing to ORFs, we also searched for differences in the nature of binding between gRNAs to canonically edited transcripts and binding between gRNAs to edited patterns within reads that are not part of a canonical ORFs. The comparison showed that both length and average number of G:U base pairs significantly differed in these two groups (Supplementary Figure S5). Alignments between gRNAs and productively edited transcripts are longer, making it likely that binding affinities of the non-cognate interactions are typically lower than those of the cognate interactions. This phenomenon is consistent with limited-length edited regions and inconsistent patterns observed in the edited reads for complex I cryptogenes that fail to assemble into patterns leading to ORFs.

### RNA editing machinery in *B. nonstop* is conserved regardless of recoding and aa insertions

In addition to direct effects on the mitochondrial genome, the nuclear genome codon reassignment could also indirectly impact mitochondrial gene expression. For instance, because RNA editing and other RNA processing factors are nucleus-encoded, their overall expression could be affected by the degree to which in-frame stop codons are utilized in these proteins. To address this question, we first determined the repertoire of mitochondrial RNA processing factors in *B. nonstop* with its relatively narrow range of RNA editing and compared it to that of *T. brucei*.

Out of 71 proteins involved in RNA editing and processing in *T. brucei* (they constitute PPsome [5′ pyrophosphate processome], MPsome [mitochondrial 3′ processome], RECC and RESC), 65 orthologues were readily identified in *B. nonstop* (Supplementary Table S2). Only six proteins were not found in the BLAST-based analyses, five of which are known to exist or be functional only in *T. brucei* or its close relatives (90–93). Additionally, *T. brucei* RESC proteins RESC11 and RESC12 emerged from a duplication in the common ancestor of known *T. brucei* strains (Supplementary Figure S6A,B); this protein is single-copy in other trypanosomatids, including *B. nonstop*. The KREPB6 and KREPB7 proteins in *B. nonstop* could be distinguished only phylogenetically (Supplementary Figure S6C). Whereas the population of individual RNA editing factors was remarkably conserved between *B. nonstop* and *T. brucei*, this generalization does not extend to the RNA processing complex, the MPsome. While the two catalytic proteins of the *B. nonstop* MPsome are clearly conserved, its six non-catalytic members share a much lower degree of sequence similarity with those of *T. brucei* than all other mitochondrial RNA processing proteins. The MPsome subunit 1 (MPSS1) is missing in *B. nonstop*, and a global alignment of MPSS3 and MPSS4 subunits reveals only about 15% identity (Supplementary Table S2). The absence and divergence of non-catalytic components implies that the overall structure of the MPsome may be poorly conserved across kinetoplastids. In terms of U-indel editing, however, it likely matters little, as the MPsome is involved in processing steps largely preceding editing (19).

Armed with this knowledge, we then addressed potential impacts of the *B. nonstop* nuclear genome codon reassignment on the identified mitochondrial RNA editing and processing machinery. In essence, the genome under study has three ‘extra’ sense codons, so their inclusion may potentially decrease the overall translation efficiency in a particular set of genes. Thus, we asked whether these codons were overrepresented in mitochondrial RNA editing and processing machinery by comparing read-through stop codon abundances within this group of proteins (Supplementary Table S2) with the abundances of the likely evolutionary predecessor codon that results in the same amino acid (9). The usage of all three recoded codons in the RNA editing proteins trended higher than that of the standard codons, although the magnitude of the difference between actual versus predicted values was small, and only for TAA and TGA were there statistically significant differences (Supplementary Figure S7). Notably, a group of mitochondrial proteins involved in a different process, namely the iron-sulfur cluster assembly, showed the opposite trend, and at a greater magnitude. However, when compared with the codon usage of proteins of a similar non-mitochondrial function, such as nuclear RNA splicing, codon usage profiles of the mitochondrial RNA processing proteins were similar (Supplementary Figure S7). Therefore, there is little (if any) evidence that the mitochondrial genome's expression is specifically impacted by recoding of the nuclear genetic code.

**Figure 8.**
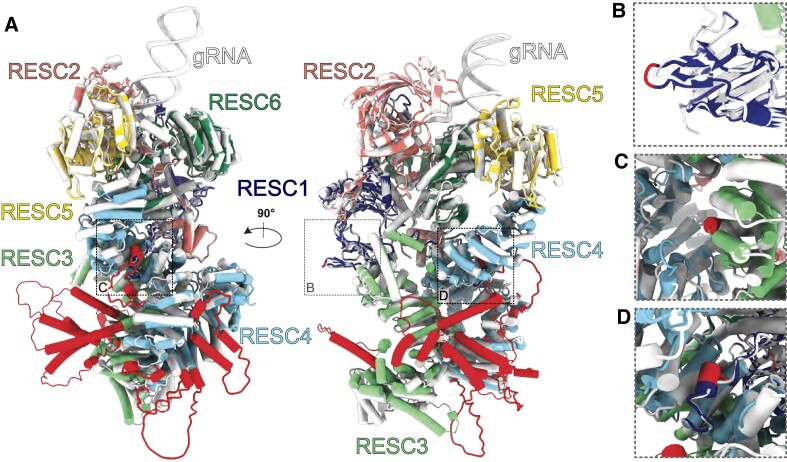
*Blastocrithidia nonstop* RESC-A model. (**A**) The *T. brucei* RESC-A cryo-EM structure (PDB ID 8FN6) is shown in white, while models of individual *B. nonstop* RESC subunits are each colored differently and superposed with the *T. brucei* structure. Insertions in *B. nonstop* homologues are highlighted in red. RESC3 and RESC4 were predicted by Alphafold, all other proteins by Swiss-Model. (**B**) An example of a surface-facing insertion of RESC1. (**C**) An example of a cavity-facing insertion of RESC3. (**D**) An example of four amino acids of a RESC1 insertion that likely contacts RESC4, extending the interaction between these subunits.

Surprisingly, the most apparent difference between the sets of post-transcriptional processing proteins between these species were their individual protein lengths: *B. nonstop* orthologues are on average 29% longer, mainly due to frequent aa insertions of an average length of 16 aa (Supplementary Table S2). The aa insertions within RNA editing proteins could affect protein-protein and protein-RNA interactions of the various complexes they participate in. To determine the likelihood of this, we predicted structures of all *B. nonstop* RESC subunits by homology- and *de novo* modeling and compared them with *T. brucei* cryo-EM structures (26). Approximately half of insertions were not mappable on the *B. nonstop* homology models because they do not occur in regions resolved by cryo-EM. In *de novo* models, these insertions were located either in the flexible regions with poor scores or in the exposed loops between secondary structure elements. Most remaining insertions in the models manifested as extensions to helices or loops positioned predominantly on the surface of RESC-A or RESC-B. The few buried aa insertions were cavity-facing. In rare instances where an aa insertion was positioned to potentially interact with another complex protein, the few aa involved were likely to increase rather than decrease interaction interfaces. Examples of the RESC-A surface, buried, and very short protein-protein interface aa insertions are depicted in Figure 8. Notably, some insertions within the RESC subunits such as those of RESC4 or RESC9 are extensions of insertions also present in *L. major* (LmjF.28.0340 and LmjF.33.1730, respectively). This indicates that variability in these regions is tolerated. We also modelled an enzymatic RECC subunit, REL1. All three of its aa insertions were in the termini not present in the solved crystal structure (thus, likely representing flexible regions) implying that the insertions do not impact the ligase activity. In conclusion, abundant as they are, *B. nonstop* aa insertions in the examined proteins are unlikely to result in functional deviation from those of the homologous complexes in *T. brucei* or other trypanosomatids, consistent with the hypothesis set forth elsewhere (94).

## Discussion

The discovery of recoding of the *B. nonstop* nuclear genome provided a unique opportunity to determine the degree of influence this genetic peculiarity has on the mitochondrial genome. Importantly, this recoding is restricted to a genus within a phylogenetic group of which the mitochondrial genome and its expression have been intensely studied (1,4). We found the *B. nonstop* mitochondrial genome and its expression to be largely insulated from the results of the nuclear genome recoding, despite the fact that unique tRNAs responsible for the read-through of stop codons of its nuclear genome are apparently not excluded from the mitochondrion (Figure 2). If these tRNAs are in the mitochondrion, it is surprising that there is not more of a ‘footprint’ of their presence on the mitochondrial genome, such as utilization of stop codons within maxicircle coding sequences. Perhaps the most likely mechanism to explain this is a modification (‘deactivation’) of the two unique tRNAs once they enter the mitochondrion rendering them inefficient in translation. There is precedent for such a mechanism. For example, RNA editing of CCA to UCA of the canonical tRNA^Trp^ in the mitochondrion of kinetoplastids allows the codon TGA to code for tryptophan rather than stop (95). Several putative cytidine deaminases were identified in the *T. brucei* genome (66) and now in the *B. nonstop* genome (Supplementary Table S5). Some of them were predicted to be targeted to the mitochondrion and thus could potentially act on these tRNAs to render them nonfunctional for a mitochondrial translational read-through role. An alternative explanation for the lack of impact of the suppressor tRNAs^Glu^ could be that they are never charged by a glutamyl aminoacyl tRNA synthetase (Glu-aaRS). This enzyme was found to be encoded by a single-copy gene in the *B. nonstop* genome and predicted to be targeted to both cytosol and mitochondrion (Supplementary Table S5) as in *T. brucei* (65). For specific tRNA aminoacylation, aaRS require a set of determinants and anti-determinants within tRNA molecules. In addition to the anticodon, these identities are often represented by specific tRNA modifications, such as thiolations, methylations, and pseudo-uridylations, just to name a few (96). Therefore, it is parsimonious to suggest that suppressor tRNAs^Glu^ undergo specific mitochondrial tRNA modification/editing events that hinder their aminoacylation status. Unfortunately, pursuing evidence for such a mechanism is not experimentally realistic, as it would require probing for unfeasible numbers of potential known individual nucleotide modifications that could be present at any number of sites on these two tRNAs.

Addressing the original question required characterization of the *B. nonstop* kinetoplast genome and its expression, which resulted in the serendipitous discovery of unusual features of its mitochondrial RNA editing and the proteins that execute it. One of the most unusual aspects of the *B. nonstop* kinetoplast RNA processing factors is their increased (compared to the same protein repertoire in other trypanosomatids) size, driven by the presence of multiple short aa insertions. The fact that so few RESC protein-RNA or protein-protein interactions are apparently disrupted by these insertions (Figure 8) suggests that these insertions are not functionally neutral. In turn, this implies that conservation of the RESC structure as well as the retaining of its component proteins is critical to U-indel RNA editing. The likely origin of the aa insertions is also worth considering. We previously reported that *Blastocrithidia* and *Obscuromonas* spp. (previously collectively named ‘*jaculum*’ clade) lack the critical Ku70 and Ku80 proteins of the non-homologous end-joining pathway, while all other trypanosomatids retain them (94). Although ablation of only Ku80 did not destabilize the genome of *Leishmania mexicana* after 100 passages (97), a direct comparison between these two species may be misleading as they are quite evolutionary distant (1). We consider the absence of the Ku proteins a most straightforward explanation for the phenomenon of unusually frequent aa insertions. In any event, they are likely unrelated to any aspect of the nuclear genome recoding.

Although the editing machinery apparently varies little among trypanosomatids, the structural and complexity variation of gRNA-containing minicircles within the group is tremendous. With only 41 minicircles, *B. nonstop* possesses by far the smallest repertoire characterized to date. The size of a minicircle repertoire influences mitochondrial transcriptome diversity because a larger minicircle population results in more gRNA loci. This, in turn, leads to redundancy of encoded gRNA coverage on transcripts, likely promoting editing to a variety of patterns, both those contributing to ORFs and those that are apparently transitional or abortive (32). Typically, three to four gRNAs capable of binding and directing editing cover any specific region of an edited domain in *L. pyrrhocoris*, and coverage is likely even greater across *Trypanosoma* spp. (24). As such redundant coverage is lacking in *B. nonstop* (Figure 7 and Supplementary Figure S4), the genetic flexibility theoretically conferred by U-indel editing in its mitochondrion may be seriously curtailed. This same lack of redundancy resulted in a high precision with which we could assign *B. nonstop* gRNAs. Only two minicircle gRNAs remain unassigned, as they bind nowhere within the fully edited mRNAs or the maxicircle read population of *B. nonstop*. For pan-edited transcripts, binding of all gRNAs (except for the ones directing editing of the initial 3′ editing sites) relies on editing of the preceding region (98,99). As loss of any gRNA within the 3′ to 5′ cascade will eliminate the sequences of all subsequent anchoring regions, *B. nonstop*’s two unassigned gRNAs may be remnants of a processing pathway for transcripts that were once edited but no longer undergo this process in the extant parasite.

It is highly notable how closely the absence of identifiable gRNAs for a complex I transcripts mirrors the loss of their productive editing. The absence of editing of the complex I subunits with parallel minicircle loss in the *Leishmania tarentolae* strain UC has been previously reported (88). Isolate UC was continuously cultured for over half a century and exhibited severely impaired editing and a loss of gRNAs for *ND3*, *ND8*, *ND9*, *G3* and *G4* relative to a strain with a far shorter cultivation history yet nearly identical maxicircle sequence. Thus, loss of editing of subunits of respiratory complexes that are no longer needed by a parasite may be a recurrent evolutionary trait. In contrast to strain UC, *B. nonstop* is a recent isolate with far <100 laboratory passages prior to kDNA and mitochondrial RNA collection. As such, we consider *B. nonstop* a natural example of specific gRNA population loss, especially as we also noted the loss and retention of productive editing for trypanosomatid *ND8* and *COIII*, respectively, in the midgut-derived cDNA libraries of the bean bug *Riptortus pedestris* infected by another isolate of *Blastocrithidia* sp. (100). However, it cannot be completely ruled out that the *B. nonstop* minicircles encoding gRNAs responsible for complex I subunits cryptogene editing were lost during even the short laboratory cultivation.

Although both the gRNAs to direct complex I cryptogene editing and the productive editing itself are absent, most complex I cryptogene-derived reads contain U indels (Figure 4). We demonstrate that the majority of these edits could be theoretically explained by brief, stochastic interactions with cognate gRNAs of other transcripts (Supplementary Figure S5). However, this explanation does not address the fact that gRNAs could also transiently bind to all maxicircle transcripts, including those of the correctly-encoded mRNAs, yet very few reads mapping to these loci contain U indels. This observation could be explained by maxicircle transcripts having evolutionarily derived editing identities of ‘permissive’ and ‘nonpermissive’. In such a scenario, gRNA and/or editing factors would only be capable of engaging permissive transcripts. Current knowledge of the roles of editing complexes do not rule out that players and/or events in editing initiation may restrict it to only some mRNA substrates. Rather than being a fixed entity, RESC appears to be a collection of proteins of which subassemblies can form (17). It has typically been conceptually considered comprised of a gRNA-binding module and a loosely-defined RNA Editing Mediator Complex (REMC) (101). While recent crystallographic structures define RESC from a gRNA-centric perspective (26), RESC may also be viewed from the perspective of REMC RNA binding proteins that interact with mRNA. These proteins may in fact identify as-of-yet unknown features of an mRNA that define it as substrate for editing. Specialized forms of REMC may exist that interact stably with permissive mRNAs and could recruit RECC with only a transient presence of non-cognate gRNA on the RESC gRNA-binding module, or even in complete gRNA absence. This will be an important point to follow as more and more maxicircle transcriptomes will become available for evaluation.

Finally, characterization of the *B. nonstop* maxicircle also revealed a surprising feature of loci of properly encoded genes. Amino acid substitution rates are unusually high in *B. nonstop COI* and *ND1*, and sequence termini truncations were observed for the *ND4* and *ND5* loci that, based on sequence alignments, are atypical. As an explanation it is worth reconsidering the two *Blastocrithidia-*specific cytosolic tRNAs fully cognate to the UAA and UAG stop codons (9) that we have demonstrated to be present to some degree in the mitochondrion (Figure 2). We speculate that unless the whole pool of these two tRNAs are rendered inactive by nucleotide modifications or another mechanism, their presence may accelerate aa sequence evolution. While this would be quite a novel way to facilitate genomic alterations, we have thus far failed to come with a more pedestrian explanation.

In summary, we demonstrated a buffering of the effect of nuclear genome recoding on the *B. nonstop* mitochondrial genome, and revealed several unexpected findings related to the kinetoplastid U-indel editing. The evolutionary forces that drove the gradual ‘deterioration’ of sequences and, ultimately, elimination of the complex I in *B. nonstop* remain to be investigated. With an ever-increasing repertoire of bioinformatic tools and opportunities for genetic manipulations across a wider variety of species, the field is well equipped to peruse this and other U-indel editing-related topics in the future.

## Supplementary Material

gkae168_Supplemental_Files

## Data Availability

Sequencing data has been deposited in SRA / BioProject under accession number PRJNA981548.

## Notes

Present address: Eva Myšková, DOE Joint Genome Institute, Berkeley, USA.
