# Supplementary material for: Blastocrithidia nonstop mitochondrial genome and its expression are remarkably insulated from nuclear codon reassignment
Source: Nucleic Acids Res. 2024 Mar 7;52(7):3870–85. doi: 10.1093/nar/gkae168 (PMC11040004; doi:10.1093/nar/gkae168)
Supplement: gkae168_Supplemental_Files [file gkae168_supplemental_files.zip › v 33 Suppl Fugures all.pdf]

BLASTOCRITHIDIA NONSTOP MITOCHONDRIAL GENOME AND ITS EXPRESSION ARE REMARKABLY INSULATED FROM NUCLEAR CODON REASSIGNMENT

Dmitry A. Afonin[1†], Evgeny S. Gerasimov[1,2†], Ingrid Škodová-Sveráková[3,4,5], Kristína Záhonová[3,5,6,7], Ondřej Gahura[5], Amanda T.S. Albanaz[3], Eva Myšková[5], Anastassia Bykova[3], Zdeněk Paris[5,8], Julius Lukeš[5,8], Fred R. Opperdoes[9], Anton Horváth[4], Sara Zimmer[10*], and Vyacheslav Yurchenko[3*]

[1] Faculty of Biology, Lomonosov Moscow State University, Moscow 119991, Russia
[2] Institute for Information Transmission Problems, Russian Academy of Sciences, Moscow 127051, Russia
[3] Life Science Research Centre, Faculty of Science, University of Ostrava, 710 00 Ostrava, Czechia
[4] Department of Biochemistry, Faculty of Natural Sciences, Comenius University, 842 15 Bratislava, Slovakia
[5] Institute of Parasitology, Biology Centre, Czech Academy of Sciences, 370 05 České Budějovice, Czechia
[6] Department of Parasitology, Faculty of Science, Charles University, BIOCEV, 252 50 Vestec, Czechia
[7] Division of Infectious Diseases, Department of Medicine, University of Alberta, T6G 2R3 Edmonton, Alberta, Canada
[8] Faculty of Science, University of South Bohemia, 370 05 České Budějovice, Czechia
[9] De Duve Institute, Université Catholique de Louvain, 1200 Brussels, Belgium
[10] University of Minnesota Medical School, Duluth Campus, Duluth, MN 55812, USA

[*] To whom correspondence should be addressed: vyacheslav.yurchenko@osu.cz (V.Y.) and szimmer3@d.umn.edu (S.L.Z.)
[†] Joint authors

SUPPLEMENTARY FIGURES S1-S7.

Cytochrome oxidase subunit I (*COI*)

```
Tbru  MFFLCLVCLSVSHKMIGICYLLVAILCGFIGYIYSLFIRLELSLIGCGVLFGDYQFYNVLITSHGLIMVFAFIMPITMGGFTNYFAPVMVGFPDMVFPRINNMSF...
Ltar  MFWLCLVCLSVSHKMIGLCYLLVAILSGFVGYVYSLFIRLELSLIGCGILFGDYQFYNVLITSHGLIMVFAFIMPVMMGGLVNYFIPVMAGFPDMVFPRLNNMSF...
      **:**************:********.**:**:**************:**************************: ***:.*** ***.*********:*****
Bnon   MWISSICLTISHKIIGLCYLFIAILCGFVGYVYSLCIRLELSVIGCGVLFGDYQFYNVLITSHGLIMIFAFIMPVIMGGFSNYFSPLIAGFPDMAFPRLNNMSF...
       :::. :**::***:**:***::***.**:**:*** ******:****:******************:******: ***: *** *::.*****.***:*****


Tbru ...WMFIGGFGCLVSGFLTEEGMGVGWTLYPTLICIDFHSSLACDFIIFSVHFLGISSILNSINVVGTIFCCRRKYFSFLIWTLFIWGALLTSILLIITLPVLAGG...
Ltar ...WMYLAGFGCVVNGFLTEEGMGVGWTLYPTLICIDFHSSLACDFVMFAVHLLGISSILNSINLLGTLFCCRRKFFSFLSWSLFIWAALITAILLIITLPVLAGG...
        **::.****:*.******************************::*:**:***********::**:******:**** *:****.**:*:*************
Bnon ...WMFVGGFICLVTGFLSEEGMGVGWTLYPTLICVDFHSSLSCDFVLFAVHFLGISSILNSINFIGTLLCCRRKYFSILSFTLFLWGALLTSILLIATLPVLAGG...
        **::.** *:*.***:****************:******:***::*:**:***********.:**::*****:**:* ::**:*.**:*:**** ********


Tbru ...VTLLLCDRNFNTSFYDVVGGGDLVLFQHLFWFFGHPEVYIIILPVFGLVSTIIEVTSFRCVFSSVAMIYSMLLISVLGMFVWAHHMFVVGMDVDSRAYFGSIT...
Ltar ...VTLILCDRNFNTSFYDVVGGGDLILFQHIFWFFGHPEVYIILLPVFGLISTIVEVIGFRCVFSTVAMIYSMILIAILGMFVWAHHMFVVGMDVDSRAYFGGVS...
        ***:******************:****:************:******:***:** .******:*******:**::************************.::
Bnon ...VTLIICDRNFNTSFYDVVGGGDLLLFQHLFWFFGHPEVYIIILPIFGLISTLIEIFCLRTVFSSISMIYSMIMISILGAFVWAHHMFVVGMDVDSRAYFGAVT...
        ***::******************:****:************:**:***:**::*:  :* ***::*****::*::** ********************.::


Tbru ...VLIGLPTCIKLFNWIYSFLFTDMCICFEIYFIYMFILMFLAGGLTGLFLSNVGIDILMHDTYFVVAHFHYVLSLGAVVGVFGGFFHFLMKWIPIELHTFWLFF...
Ltar ...ILIGLPTCVKLFNWIYSFLYTDMIITFEVYFVIMFIFMFLIGAVTGLFLSNVGIDIMLHDTYFVVGHFHYVLSLGAVVGFFTGFIHFLAKWLPIELYLFWMFY...
        :*******:**********:*** * **:**: ***:*** *.:************::*******.*************.* **:*** **:****: **:*:
Bnon ...ALIGLPTGIKLFNWIYSFFFVDLLLHIESFYIFCFIFMFLMGGVTGLFLANIGIDIMMHDTYFVVAHFHYVLSLGAVFGFFAGFFHFINKWINLEINLFFFWI...
         ****** :*********::.*: : :* :::  **:*** *.:*****:*:****::*******.***********.*.* **:**: **: :*:  *:::


Tbru ...FISTLWFGSNMVFFPLHSLGMFAFPRRISDYPISFLFWSAFTLYGMLLLTFLVIFCCCLFNVILFWDYCLFFINLFTYSLSIFFYFYTWVPVCMAIYLLVIDF...
Ltar ...FISTLFIGSNMLFFPMHSLGMYAFPRRISDYPVSFLFWSSFMLYGMLLLASLILFLCALFCVFLFWDYCLFFVSLFVFSLYCFFYFSTWLPCVMVLYLLLVDF...
        *****::****:***:*****:**********:******:* *******: *::* *.** *:*********:.**.:**  **** **:*  *.:***::**
Bnon ...FLINLFFGANMIFLPMHSYGLYGCPRRITDFPICFIFWNSVILFGILLITFVVLFLSVFFSYSFLCNACLCLDFWSSFNLDIFVTVN--IQIVSVLYLILVDF...
        *: .*::*:**:*:*:** *::. ****:*:*:.*:**.:. *:*:**:: :::* . :*   :: : ** :     :.*  *. .   :    .:**:::**


Tbru ...AHIILDYLLIILCFCFVFYIFFWQAFLLFFYI
Ltar ...AHIVLDYLFLILCFCFVFFIFFWQSLFLFFYI
        ***:****::********:*****:::*****
Bnon ...LHLCVSYLFVFLNILSVFLVSFCHFICNFLI
         *: :.**:::* :  ** : * : :  *:
```

NADH dehydrogenase subunit 1 (*ND1*)

```
Tbru  MLLLHLDICILIVIFILVLSVLCGYVSLCERKILAIVQFRIGPALFLFGLLTPITDGVKLFVKFTLFVIGVDSILFISSLFITVFCIFFPWFFFPLGFIIIFDKG...
Pser   MFFFIDLFIVILIIIILVVLILCGYTTLCERRILAIVQLRIGPALFFFGILTPITDGIKLFLKFTLFVVGFDGIYLIIALLVTTFCMFIGWLFFPIGYIILLDNG...
Ltar    MLNIDIIIIIIIDILVVLILTGFVSLCERRILALVQIRIGPALCFFGILTPITDGIKLFIKFIIFVISFDIIYLIGAMIITACCIFLGWFYFPIGFILLLDTG...
        :: :*: *:*:* ***: :* *:.:****:***:**:****** :**:*******:***:** :**:..* * :* ::::*. *:*: *::**:*:*:::*.*
Bnon    MILIDFFFIILFFVLIILILTGYFSLCERKVLAIVQMRSGPNLCFFGILTPITDGIKLFLKFVNFVVNVNAILLFLMLFSWFFVNLSVIFMLPFGLFTVLPKI...
        :: :*: ::*:: :*:: :* *: :****::**:**:* ** * :**:*******:***:**  **:..: * ::  ::      :   : :*:* : :: .


Tbru  ...FTLLFLLGFHLFSNVFCIFFVGCFLFSSCFIYLAAMRTLFFSILSECSILILLYCIYILDYFCFFGIKDICISQLSLQNCFILGLLFICLFWIGLLLDGLRL...
Pser  ...FTVFLMLFLHILINVCSVFWVGFFLFSSCFVYMSAMRVLFFSIISESSMLMLFLISFLLDNFSMLSIKDVSIGQLYINNFFLGGILFVGLFWICLLIDGLRI...
Ltar  ...FTLTVMLCVHVFCSMFSTFFVGCFLFSSCFVYLSAMRTMFFSIISESGIFLLYTTIYSLDYFSFFGIKDVCVGQIYITNFYIAGVLFISVFWVSMLLDGLKL...
         **: .:* .*:: .:.  *:** *******:*::***.:****:**..:::*    : ** *.::.***:.:.*:: * :: *:**: :**: :*:***::*
Bnon  ...FNIFIFFLIILLCDLCLAIFVGYFLVTSVYAFISCSRLLLLILSSESLILHFIIVMFLIQINSYCNIAYIIISQFSFYNFFFCGIFIFMFFLVWFLLECKKL...
         *.: .:: . :: .:   ::** **.:* : :::. * ::: : **. :: :    : ::  .  .*  : :.*:: * :: *:::. .* : :*::  ::*

Tbru  ...PFDYLECESELVAGLVTELSGVFFVIYSILEINHLLLTTILFSCLCFGGLFICFKSILILILGFLIPRVICCRLKITTAQTFILLFLFTMGFINFSFIAITK...
Pser  ...PFDYMECESELVAGLITELSGFFFVIYSILETSHLLLSTILLACFCFGGLFVCFKSLIILIVCFFFPRVIGFRLKITTAQTFIILFLFIMCFLLFNWLAITK...
Ltar  ...PFDYMECESELVAGLITELSGFFFVLYSVLEINHILLTTLLFASLCFGGLFICFKAILILIFGFFYPRVIGYRLKITTAQAFILIFLFYMYVLMFIWLFTTK...
         * **:**********:*****.***:**:**. *:**:*:*::.:******:***::***. *: ****  ********:**::*** * .: * ::  **
Bnon  ...PFDYVECESELIAGVFTELSGIFFVISSVVEIWSSVCFSIIFVGSFFGGFFIFVKLVLFIIGFILLFRVLCFRVKITDVFFLLFFLIYWICLFFIISILIFS...
         ***:******:**:.*****.***: *::*     :  ::::    ***:*: .* ::::*  ::  **:  *:*** .  :::::: : .: :  :   .


Tbru  ...IICILF
Pser  ...LISLCF
Ltar  ...IIAMLF
         :*.: *
Bnon  ...CVFLL
          : :
```

NADH dehydrogenase subunit 2 (*ND2*)

```
                                                                          Truncated N-end
Tbru MFLYIIHIFIFLIIYSFIILCDYTTLTLLSFDLLWLLIINLFWITLLDSYICFIFILLFLFCFTLFFCFLSFDTRFLFIIIIIQYIIIFLFIFINHIIIISILFEI...
Pser MFLYIIHLLIFLFAYSFLLLCDSNILHYLSFDLFWINIINILLLFTIDSYICFIFFFLFSFIYGLTHTFATFDLRMLLFIVMIQYVCIFMFLYVIHILYISILFEI...
Ltar MFLYLIHIILFLLLYSFIILCDYTSLFYLSFDLIWLIIINIIILTILDSYICFIFLLLFLFCFFFLFCFLNFDTRFVFMIIIMQYIIIFMFLHVIHILFISILFEL...
     ****:**:::**: ***::*** . *  *****:*: ***:: :  :*********::** * : : . * .** *::::*:::**: **:*:.: **: ******:
Bnon                                   MFSRKLSYIFLFVFFVWMYSYFFIIFLFFIAYCAPHTTSSLSNSLIMLQFLAVTLANCTSLIVLFLFFFEI
                                       :   ::  :  :  :: :   ::: * : : .         : ::::*:: : :      *: : ::**:

                                                                                 Gap
Tbru ...FSLLLFLLLMSSRFGYKILVLWYYYMLNLINFILLFILLYFMILNYCFFLCDFCFLVFDEEWLGILCLFYTLLILFKLYIAFLILFMEQLYIRLGVFIFIYML...
Pser ...FSLLVFLLLISSRFGYKILILWYYYMINLISFLLLIILLYFMVLNYCYYLCDFCFLIFDEEWLGILCLMYIFLITLKLYTSLLITYMEQLYIRLGIFIFIYML...
Ltar ...FSLLLFLILISSRFGYKILILWYYYMINLINFILLFVLLYYMILNYCFYLCDFCFLIFDEEWLGILCLFYILLILFKLYIAILILFLEQLYIRLGVFIFIYML...
        ****:**:*:*********:******:***.*:**::***:*:****::*******:***********:* :** :*** ::** ::********:*******
Bnon ...FNFSVLFYIIKNQVVQNTVQIWNWFLVDLVILFFCLFFVYSSSCKNVFFYVEINFFVS------WFFYFCLFIFWCKLYFAFIFFFFSQIYLKIHKTVIVFNF
        *.: ::: ::..:.  : : :* :::::*: ::: :.::*     :  ::  :: *::       :  :  :::  *** :::: ::.*:*:::   :::: :

                                                                                                                  Gap
Tbru ...TFYILFCFILIILLISFIYFYILFIKLLLFQSCTCVLIGLNSFAIVSLLFVLSVNNFCFLFLIFISTKNYIFYLYLNFHLIYSISLVLLIIIYYFFIIYNIFD...
Pser ...TFYVLFCFILIIIFINFIFFYIILIKLAIFQCCFCVLIGLNSFAIVTLLFVLSVNNFCFLFLVFISAKNYVFYLFLNFHVIYSCSLVLLLLLYYFFIVYNMFD...
Ltar ...TFYVLFCFILIILLICFIYFYIIFIKLIIIQSITCVLIGLNSFAIVSLLFVLSVNNFSFLFLIFISTKNYIFYMYLNFHLIYSLSLIILIILYYFFIVYNMFD...
        ***:********::* **:***::*** ::*.  ************:**********.****:***:***:**::****:*** **::*:::*****:**:**
Bnon ...TIYIFFISTWITYFTILNVFISLITKFCFIHTFFAFLILLSMHDWISIFFINSCINN--IFLLIFLFKSFILYLWFFFHILYSICIIFFFFLSASFNSG----
        *:*::*    *  :  :  *  :: *: :::   ..** *. .  ::::*: *  *   :**:::  *.:::*::: **::** .:::::::   *

        Gap
Tbru ...FKYNENYFLINFIFFSFFNNFLISLLLACLFLCIGAIPIVFGFFIKVFCLLLQLSYLCICIGFFFIIWLIIIYIFYFRLIVNIFIFSYQFLGFWVVKLSFINI...
Pser ...IKYNENYFLLNYVFFSFFNSLVLSVFLACLILCIGSIPITFGFFLKTFGLLLQLSALGISILFFSIIWLTITYMFYFRLIVQIFIFSYQFTGFWVVRLYTLTT...
Ltar ...IKYNENYFLINFIFFSFFNNLLISIMIACIFLCIGSIPIVFGFFLKVFCLLLHLSYLGICIVFFSIIWLIIIYIFYFRLIINIFIFSYQFIGFWVVRLHILGF...
        :********:*::*****.:::*:::**::****:***.****:*.* ***:** * *.* ** **** * *:******::******** *****:*   :
Bnon ...-----------------FMCYAHNLIIFLILFICSSAPLTSIYFLKMFYFIFFVIDLSQVFLYN---ICCLYVFFYYNYLVLFFNSHRHFFLFFRYKSMKIFF...
                         *     .:::  :::   .: *:.   :*:* * ::: :   *   : :       :  :**:. :: :*    :*  *:  :   :

Tbru ...KNLLFFICSSVYILFFDIINLFDLIL
Pser ...LHLLFYISILLWILFFDIINFFDLIL
Ltar ...SNLFFILSFSIFILFFDIINLFDLIL
         :*:* :.  ::********:*****
Bnon ...VCLYISVNLDFIYLLEVC
          * : :   .  *:
```

NADH dehydrogenase subunit 4 (*ND4*)

```
                                Truncated N-end
Tbru  MLKLNLICINFILLIVTIIYIYINYSFCIGIEINYVYVNIYLNYISLWFVFFMGIIMYILIFLLSKKCVSYNKYFYIVMIYMYIYINVVLIIILDDFMCFMIAFE...
Pser  MFKFFIVFLNFIILFLSICLLSINFSYCITIDIYYVYIVFLVSYIHLWFIFFMGLILLFIIFLLTRRIASYEKYFLILSTYIFIYLAVVLIILSDDFIIFMILFE...
Ltar  MFKFILVICNFILLFLIVTLIFINYSFCLALQFNYIYINIYLNYINLWFIYFMGLIVFFLIFLLSRKLVSYSKYFYILLSYIFIFFDVTLIILIDDFMCFMILFE...
      *:*: ::  ***:*:: :  : **:*:*: ::: *:*: : :.** ***::***:*: ::****::: .**.*** *:  *::*:: *.***: ***: *** **
Bnon                               MDLVFFYCVINMNYIYILYIIGILCINSIIHFYIWQNNYNIEKKKLFSTCILIIVICILLTLFCRDVFFFLIIYE...
                                   ::: :.*  : :.** : ::  :  *  :: * : :.  . .*   :    : * : : * ::  *.: *:* :*

Tbru  ...SLFFPICLVSLFFNFNNRFIFAIFYLIIFSSVSSVVCIIICIIVISHFNIINLQAFIDVCYFDSLYSAIFIWILLFIMFAIKYPIWPFHVWLPEMHVEVNTE...
Pser  ...SLFFPICFVSLFFNFNNRFIFAIFYLIIFSSISSVLCVLICVLVIAHFNVLSLYLFTDLCFLDSLTTIIFLWVCLFVMFAIKYPIWPFHVWLPEMHVEVNTE...
Ltar  ...SLFFPICFVSLFFNFNNRFIFAIFYLVIFSSLSSIMCIIICIIIIFHFNILNLQSFIDICIFDSLYLGLYVWILLFIMFSIKYPIWPFHVWLPELHVEVNTE...
         *******:*****************:****:**::*::**:::* ***::.*  * *:* :***   :::*: **:**:**************:*******
Bnon  ...LSFLPIVIGFFWFNTANRLLISIYYLFIVNTMTSILTAIFCCCFCVFFNFVFFS--NNLCLHN--TISCFLILLLFLIFSLKYPIFPFHWWLPEVHVEASTE...
           *:** :  ::**  **::::*:**.*..:::*::  ::*  .  .**.: :    ::*  :       :: : **::*::****:*** ****:***..**

                         Gap
Tbru  ...MSVLLASIVLKIGFFGVYKFLFIAFNTISIWFLGFIDSVIVLGLVFIAMSLIFLSDYKKIIANWSIIHTGIGLILLWHNDILFVGLLILCNLAHILSSSFMF...
Pser  ...MSILLASIVLKIGFFGVYKFLFLNALQVSIWFLGFIDSIIVLGLTFLSILLLFIPDYKKIVANWSVIHTGIALILLWHNDLLFIGLVFLCNISHILSSSFMF...
Ltar  ...LSILLASVVLKIGFFGLYKFLFLSFNQLSIWFLGFIDSLVMLGLTFLAITLLFLSDYKKIIATWSVIHTGIGLILLWHNDILFLGLLIFCNLSHIISSAFMF...
         :*:****:********:*****:    :*********::***.*::: *:*:.*****:*.**:*****.********:**:**::**::**:**:***
Bnon  ...ISIVLASVILKVGFYGFLKFFVFEP------FLFLICLLVLLSFVFFFWHILFFFDYKKLVAYLSIFHTNLAIILVFSYENLCILIFCIGNFLHIFTSFCLF...
         :*::***::**:**:*. **:.:       ** :*  :::*.:.*:   ::*: ****::*  *::**.:.:**::  : * : :. : *: **::*  :*

Tbru  ...IVIGYMYDNYGVRIFLLLISFFGISIWSSLFLCLFLFNIDFPFMLLFYVDIFILYGLISISFIYIISFYIITLTIFLSSIYIYMCLSFYSFVWLDKYLRLDV...
Pser  ...LVIGYMYDNYGVRVFLLIVSFFGLSIWSFMFLSIFLYNIDFPFMLLFYVDIFVFYGLISLSCIYIICFYIVVLSVFVSSIYIYICLSFYSFIWLDKYLRLDL...
Ltar  ...MMVGYMYDNYGVRIFLMLISFFGISIWSSLFLGIFLFNIDFPFMLLFYIDIFLLYGLISLSFIYICCFYIIVLAVFLSSIYMYMCLSFYSFIWLDKYLRLDL...
         :::**********:**:::****:**** :** :**:***********:***::*****:* *** .***:.*::*:****:*:*******:*********:
Bnon  ...FFFGWFYSNNGFRSNITFFNVICNNFYCFFSYLILLINIDFPFFVGFIVEILCVSQLLNVSSFFCFWVLCCIYLLFVCAVWLFCCINYTAEFWRNNFLKVDF...
         :..*::*.* *.*  : :...:  .::. :   ::* ******:: * ::*: .  *:.:* ::    .       :*:.:::: *:.: : .* :::*::*.

Tbru  ...SINDIYVFMSISISTIVFYYFIYLLI
Pser  ...SINDVYLFFITAGMTLSFFYIFYIIF
Ltar  ...TINDIYLYFITSIIVILFFYLIYLLF
         :***:*:::   :   .: *:*::*:::
Bnon  ...SINECFIILFNIIILIFFFFFTYYIF
         :**: :: :       : *::: * ::
```

NADH dehydrogenase subunit 5 (*ND5*)

```
                                                                              Truncated N-end
Tbru MFLIFFLFFIMFGFISGSFMFGRNFLSFWLSLVMIIFIVLCMIFSFLMVSVCLYGYYYYDFCLILMLDFCFIWLTYVCSGFYMFIMLLINMVFCFIVFYAFYYMY...
Pser MFLFFLLLFFMFGMLSGTFVLGRLVWSFWLEMLVQMCIFLINFTYFFCIPVCIYGYTFFDFCLLFTLDMFSIQLVFFLNGFYIFILFMINLIFCFIVFYALYYMF...
Ltar MFYCFFYVFFMFGFICGIFFIGRHILSFWLSVVLCIFLVMSTIFSCFCLSICIYGYCFYDFCLLIMLDFCFIWLVFYCNGFYIFILYLIDIVFCFIVFYAFYYMY...
     **  *: .*:***::.* *.:** . ****.::: : :.:  :   : :.:*:*** ::****:: **:  * *.:  .***:**: :*:::********:***:
Bnon                                         MVCKKIFTLTVFLCNARIYNFVFLLVFN-----------SKTLFICTCILICFDTIIFYTTFYFF...
                                              : :.: : .   ::* ::: ::         .  :**   * : *  *:**: :*::

                                                                                                  Gap
Tbru ...FDMLLGRFLIIFWIFVVCMNLFILSYDFLTAYCGWELLGLFSFFLISYFWYRFFALKFGFKAFFIGKIGDVLLIFAFSIIFLSNGFCMTTFYFLNFFCMDYY...
Pser ...FDTFLYRFFVVFWLFVLCMNFFVLAFDFITAYCGWELLGLFSYFLISYFWFRFFALKFSFKAFFISKIGDVTFLITILLCIYTNGYSCITYHLVTFFNVDFF...
Ltar ...FDMLLARFFHIFWWFVLCMNFFILSYDFLTAYCGWELLGLFSFFLISYFWYRFYALKFGFKAFFISKVGDVLLLLVFAITFLMNGYCVITFYFLTFLCVDYI...
        ** :* **: :** **:***:*:*::**:************:*******:**:****.******.*:*** :::.: : :  **:.  *::::.*: :*:
Bnon ...FSCFFFTFCIELLWFAFTMVFFVFFGNFFSIFVSWEFLGLQSFFLVNFNLSRFLCSRNSIKVVLVNKTGDIFFLIVISLWIKICMSFSS-----QVLYLDNL...
        *. ::  *   :  *.. * :*::  :*:: : .**:*** *:**:.:   ** . : .:*..::.* **: :::.: : :        .: :*

                                                                            Gap
Tbru ...YIEFSICLLVGCAFTKSTQFGLHIWLPDAMEGPIPVSALIHAATLVVCGIILLSFVYWCFDFWFSYFYNLIGWSTLILILMTLCVFYNFDVKRYVAFSTICQ...
Pser ...FIVWALFMLLMCAFTKSTQFGLHIWLPDAMEGPIPVSALIHAATLVICGIVLVSFLFWCYDFWFCCFYPILYWASLILVVMSMSVVFNFDVKRYVAFSTICQ...
Ltar ...FIVFIVFLLLTCGFTKSTQFGLHIWLPDAMEGPIPVSALIHAATLVVCGIILISFVFWCFDFWFCYFYSLIGWSSLILVMMSLCVFYNFDVKRYVAFSTICQ...
        :* : : :*: *.**********************************:***:*:**::**:****. ** :: *::***::*::.*.:**************
Bnon ...HYFAIFLFLFLCCITKSAQFAFHIWLPDAMEGPIPVSSLIHAATLVLCGIILLDKLLWLN---HHTSFNFLWWFWLLGILIVTKVHMQFDLKKIIAYSTLCQ...
        .    . :*. * :***:**.:***************:********:***:*:. : *     .   : :: *  *: :::   *  :**:*: :*:**:**

Tbru ...ISFSMFCCLCIDIYIGSLFFCYHMFYKATLFIVLGIWIHIFFGLQDLRCYFFMYFCGCVLARLLLIFAILNSCSIWFLCGFYCKDMLLALLMLLSFYNIIEF...
Pser ...ISFSMFCCLTVDIYVGALFFCYHMFYKAALFIIIGVWIHFFYGLQDVRCFFFFHFCGCILAKVLVVFALFNSCSFWFLCGFYCKDFLLTYMFIASTTYFYEF...
Ltar ...ISFSMFCCLSLDLYVGCLFFCYHMFYKATLFIVLGVWIHFFFGLQDVRCYFFTYFCGCILARMLLIFAILNSCSLWFLCGFYCKDLLLCLLMLTSFFFILEF...
        ********* :*:*:*.***********:***::*:***:*:****:**:** :****:**::*::**::****:**********:**  ::: *   : **
Bnon ...INLSFLFLFFFDSTFCYLFFANHLFYKSSLFLVIGIILHWSNNNQNFKFIHNLFKWQFMFCKILLCFVLLQSVSYWVCFGFVCKEVVLINSQWTN-ILNLEF...
        *.:*::  : .*  .  ***. *:***::**:::*: :*    . *:.:  .  .    ::.::*: *.:::* * *.  ** **:.:*       .     **

                                                      Truncated C-end
Tbru ...LFISIIFIFFTMIYNYFLLFFLMFVFKCFCLVDCLFLLFDYECCLVYCLISLYMCILSIFFIIDFVCIFVFSSYCVFWSFFLNFYNFFDIAIFVVFLILSVG...
Pser ...LIVSILFVFITIIYNYYLLFFFCFVFKCFCLNDCLFLLFDFECCLIYCFFCVYLCFIMLFFVIDFLCVFIFSSFCFFWMFFSNFYNFFDIAFFVIFMILCSA...
Ltar ...LCVCLFFIFFTVIYNYFLLFFLCFVFKCFCLVDTLFLLFDFECCLVYCTFCLYMCFVLLFFVLDFLYVFIFSSYCLFWSFYLYYASFFDIAIFTVFIMILLS...
        * :.::*:*:*:****:****: ******** * ******:****:** :.:*:*:: :**::**: :*:***:*.** *:  : .*****:*.:*:::  .
Bnon ...IFGFFFFFLGNVFYNFFILLILCFTHKNSNNFLSVFFLKMRLYCYTFFEI
        :   ::*.: .::**:::*::: *..*        :*:*     *  :  :

Tbru ...FLYYGCLFFYFFNIDCIMLFWRIFFVIIILVVFMIFCCWYFVCMIIFMLLFVWNFVIYFRYNLKYCLFFCILWILYV
Pser ...FLNYGNSFLFLFSNDCINFFWKIFTIFVFILLFSLFYCWYFICFFIYIVFFVWNIVIYFRYNFKYCLFFCIVLLFFI
Ltar ...FIYYGCVIFYFFNIDCIMFFWRVFLFITMGFLFFIFTTWYFICFYMYICMFIWNLVIYFRYNLKYCLFFCMLFIIYI
        *: **  ::::*. *** :**::* .: : .:* :*  ***:*: ::: :*:**:*******:*******:: ::::
```

Fig. S1. Protein sequence alignments of never edited transcript products (*COI*, *ND1*, *ND2*, *ND4*, and *ND5*) for *Trypanosoma brucei* (Tbru), *Phytomonas serpens* (Pser), *Leishmania tarentolae* (Ltar), and *Blastocrithidia nonstop* (Bnon). *COI* is not present in *P. serpens*. *Blastocrithidia nonstop* sequences are in bold. Sequence identity is depicted below the alignments. The conservation line output in the Clustal format: '*' indicates positions which have a single, fully conserved residue; ':' indicates that one of the following 'strong' groups of aa is fully conserved: STA, NEQK, NHQK, NDEQ, QHRK, MILV, MILF, HY, FYW; '.' indicates that one of the following 'weaker' groups of aa is fully conserved: CSA, ATV, SAG, STNK, STPA, SGND, SNDEQK, NDEQHK, NEQHRK, FVLIM, HFY. Specific features (gaps and truncations) are highlighted in shades of red.

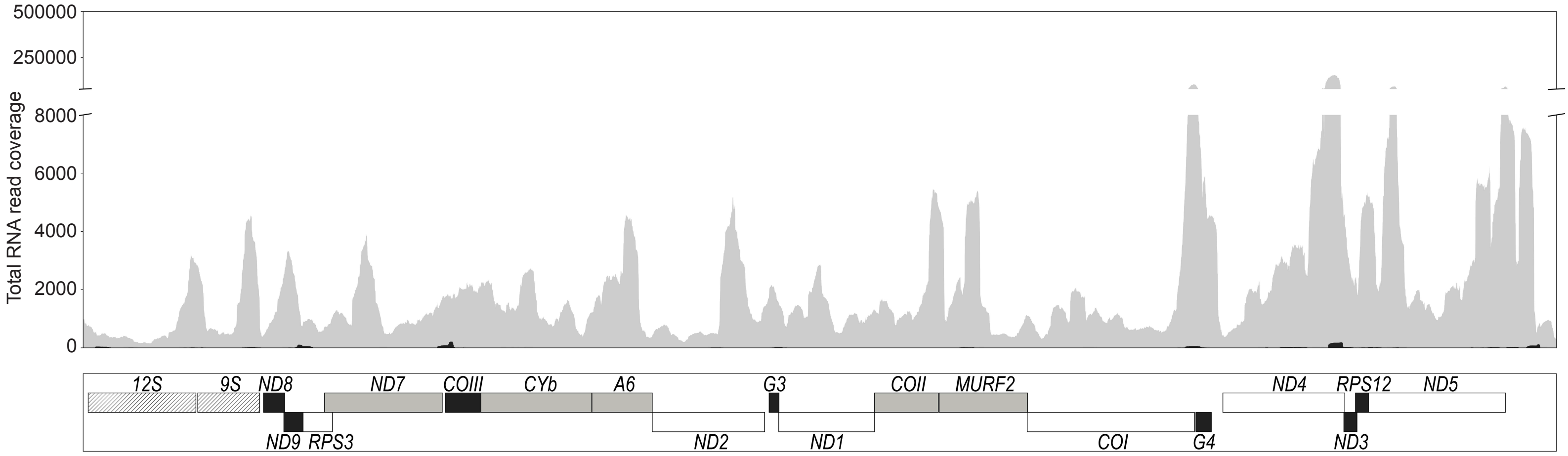

Fig. S2. Transcription and editing profiles of *B. nonstop* maxicircle with kRNA-enriched libraries that were not poly(A)-selected during preparation. The number of detected reads is shown on the Y axis in grey. The number of edited reads (with ≥5 edited sites) is shown in black. The schematic organization of the maxicircle conserved region is shown below.

***Cyb* (cytochrome b) cryptogene**

```
...A-GAAGA-A---CGTA--AAA-----AA----------G--A---AAAAA---A-G--CTCTTTTAACATCTGGGTGTTTATTGCGAATTTACGGCGTGGGA...
   UGUUUUUAUAAAAUAUAACUUGAGAAUUAAAAAAAAAACGAAAAA < mx2
   |::||:||||||#:||||#||:|:|||||||||||||||:|#|||
                                  AAAAAAAUAAUAAACUUUUGAAUGUAAGAGAAAA < mx1
                                  |||||||:||||||#||||:|||::|||||||||
...AuGAAGAuAuuuCGUAuuAAAuuuuuAAuuuuuuuuuuGuuAuuuAAAAAuuuAuGuuCUCUUUUAACAUCUGGGUGUUUAUUGCGAAUUUACGGCGUGGGA...
   M  K  I  F  R  I  K  F  L  I  F  F  L  L  F  K  N  L  C  S  L  L  T  S  G  C  L  L  R  I  Y  G  V  G  ...
```

***COII* (cytochrome oxidase subunit II) cryptogene**

```
   ...TTAGGTATTAAAGTAGA--G-A-ACCTGGGCGATGCAATGAATTAATAGTATTAAATACAACATCATCTACTGTCTATGGTCAGTGCTCAGAATTGTGT...
                3'-...UGAUAUAUGGAAUU
                      |:|:|||||||#::
5'-...UUAGGUAUUAAAGUAGAuuGuAuACCUGGGCGAUGCAAUGAAUUAAUAGUAUUAAAUACAACAUCAUCUACUGUCUAUGGUCAGUGCUCAGAAUUGUGU
   ...L  G  I  K  V  D  C  I  P  G  R  C  N  E  L  I  V  L  N  T  T  S  S  T  V  Y  G  Q  C  S  E  L  C  ...
```

Fig. S3. Editing reconstruction of minimally-edited *CYb* and *COII* cryptogenes. The top line in each panel represents the cryptogene DNA sequence. Under the DNA line, the gRNA sequences (in 3'-5' direction) are displayed, aligned to the corresponding positions of the mature mRNA. Inserted nucleotides are shown in light-red lowercase (u). Vertical lines '|', colons ':' and hashes '#' in alignments represent Watson-Crick pairs, G:U pairs and other mRNA:gRNA interactions, respectively. *CYb* is edited with two maxicircle-encoded gRNAs (mx1 and mx2), while *COII* is edited by single *cis*-encoded gRNA. The stop codon (UAA) for *COII* is highlighted in green. The encoded amino acid sequence is presented under the row of mature mRNA (at the bottom of each panel).

***COIII* (cytochrome oxidase subunit III) cryptogene**

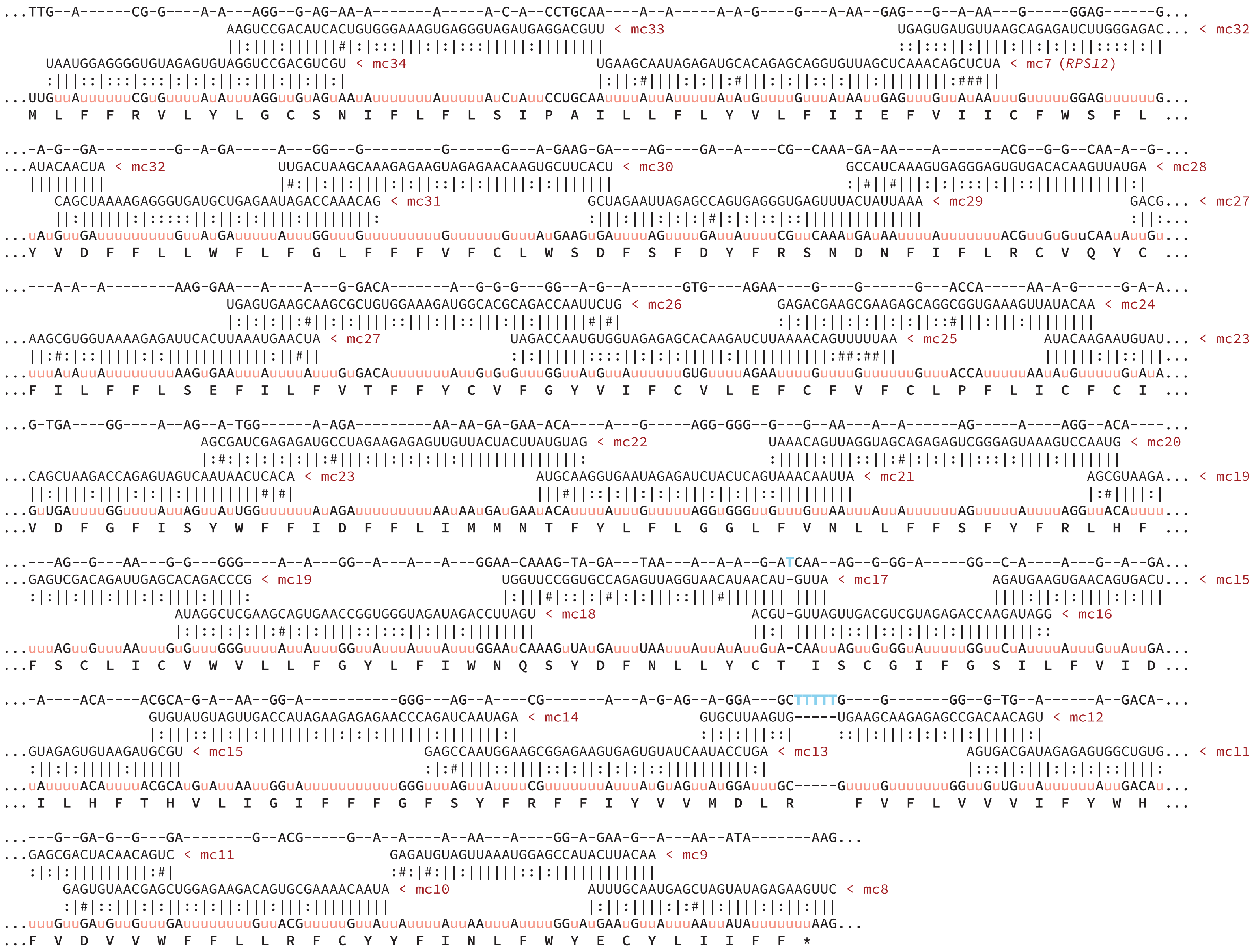

Fig. S4. Reconstruction of the cascade of gRNAs required to direct editing of the pan-edited *COIII* cryptogene. The top line in each panel represents the cryptogene DNA sequence. Under the DNA line, the gRNA sequences (in 3'-5' direction) are displayed, aligned to the corresponding positions of the mature mRNA. Inserted and deleted nucleotides of RNA are shown in light-red lowercase (u) and blue (T), respectively. Vertical lines '|', colons ':' and hashes '#' in alignments represent Watson-Crick pairs, G:U pairs and other mRNA:gRNA interactions, respectively. The label "mcN" refers to an aligned gRNA, where N is the number of minicircle encoding a particular gRNA (Table S4). The encoded amino acid sequence is presented under the row of mature mRNA (at the bottom of each panel).

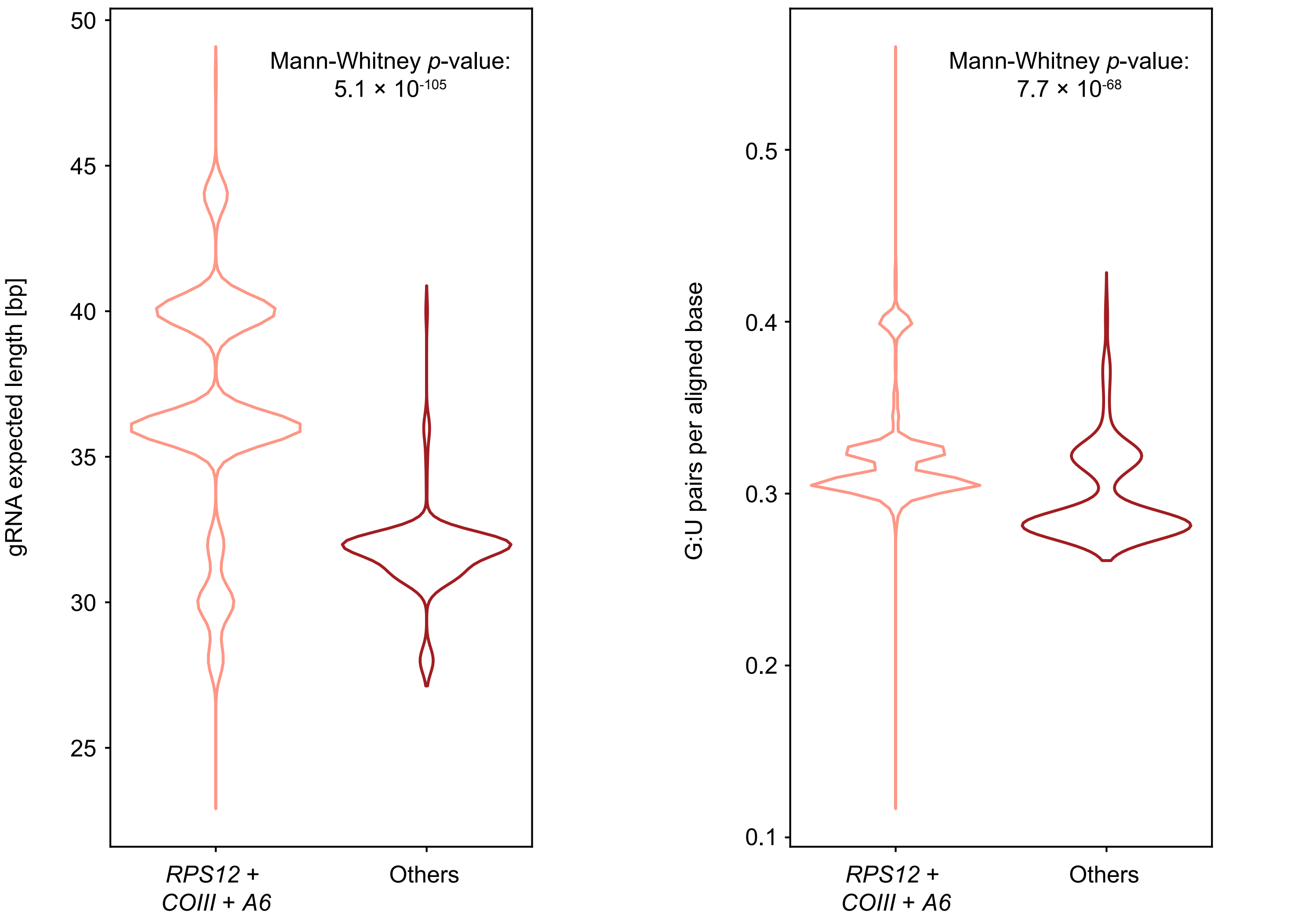

Fig. S5. Violin plots showing distributions of gRNA length (left) and proportion of G:U pairs (right) for gRNA:mRNA alignments stemming from productively edited *RPS12*, *COIII*, and *A6* cryptogenes (light-red), and from edited reads mapped on non-productively edited *ND3*, *ND8*, *ND9*, *G4*, and *G3* cryptogenes (termed 'Others') (dark-red).

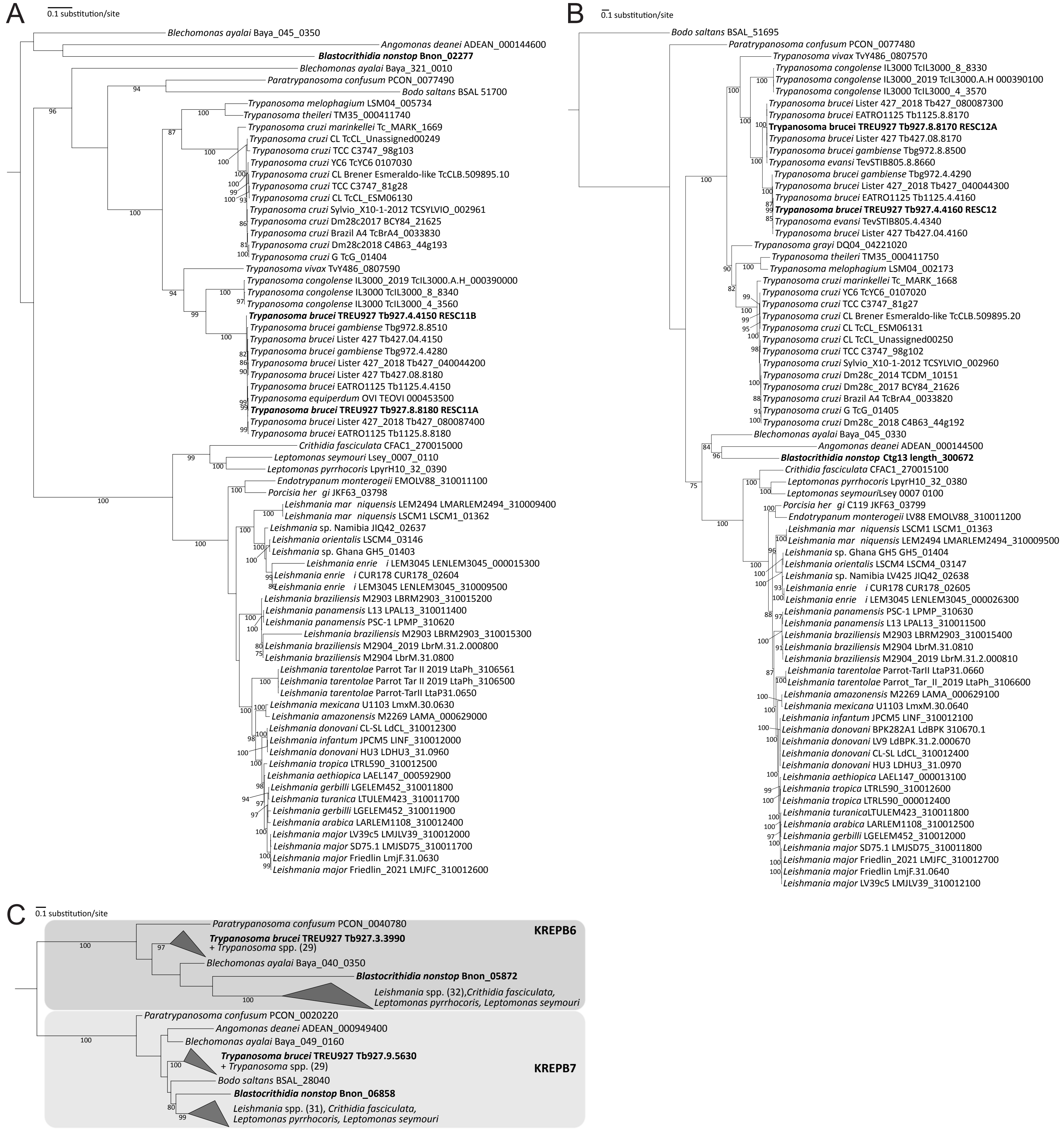

Fig. S6. Maximum likelihood phylogenetic trees of RESC11 (A), RESC12 (B), and KREPB6 and KREPB7 homologues (C). *Trypanosoma brucei* and *B. nonstop* sequences are in bold. The trees were rooted at midpoint and only ultrafast bootstrap supports over 75% are shown.

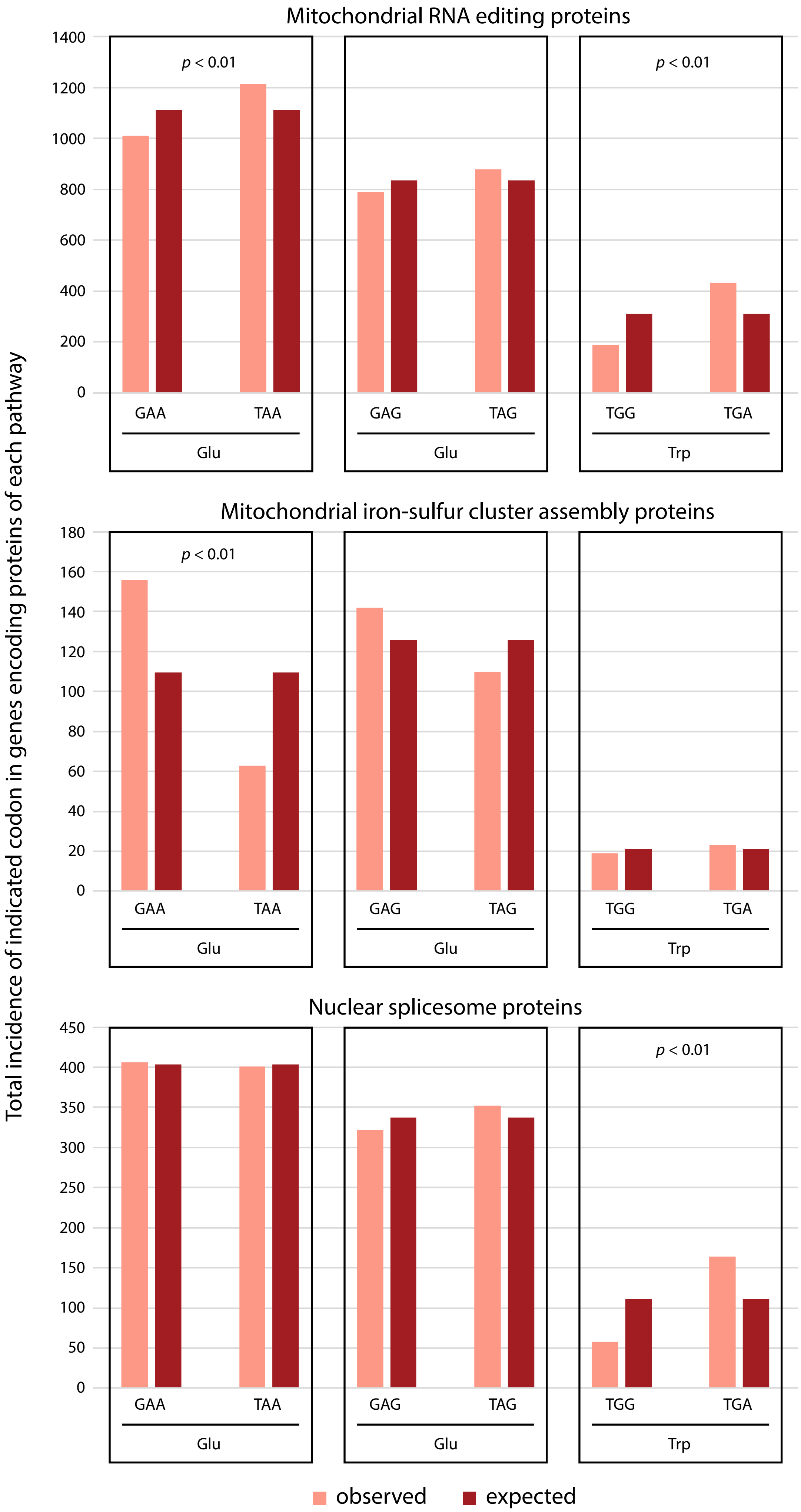

Fig. S7. Incidence of recoded codons in genes encoding proteins of a mitochondrial RNA processing complex (RNA editing proteins), a mitochondrial complex unrelated to gene expression (iron-sulfur cluster assembly), and of a non-mitochondrial, RNA processing complex (spliceosome). The differences between the observed and expected numbers were tested using the χ^2 test separately for the GAA–TAA, GAG–TAG, and TGG–TGA pairs. The *p*-values are shown for only statistically significant differences.
